# The Biology and Biochemistry of Kynurenic Acid, a Potential Nutraceutical with Multiple Biological Effects

**DOI:** 10.3390/ijms25169082

**Published:** 2024-08-21

**Authors:** Luana de Fátima Alves, J. Bernadette Moore, Douglas B. Kell

**Affiliations:** 1 The Novo Nordisk Foundation Center for Biosustainability, Technical University of Denmark, Building 220, Søltofts Plads, 2800 Kongens Lyngby, Denmark; 2 School of Food Science & Nutrition, University of Leeds, Leeds LS2 9JT, UK; j.b.moore@leeds.ac.uk; 3 Department of Biochemistry, Cell & Systems Biology, Institute of Systems, Molecular and Integrative Biology, University of Liverpool, Crown St., Liverpool L69 7ZB, UK

**Keywords:** kynurenic acid, SLC22A6, SLC22A8, ABCC4, oxidative stress, cytoprotectant, nutraceutical

## Abstract

Kynurenic acid (KYNA) is an antioxidant degradation product of tryptophan that has been shown to have a variety of cytoprotective, neuroprotective and neuronal signalling properties. However, mammalian transporters and receptors display micromolar binding constants; these are consistent with its typically micromolar tissue concentrations but far above its serum/plasma concentration (normally tens of nanomolar), suggesting large gaps in our knowledge of its transport and mechanisms of action, in that the main influx transporters characterized to date are equilibrative, not concentrative. In addition, it is a substrate of a known anion efflux pump (ABCC4), whose in vivo activity is largely unknown. Exogeneous addition of L-tryptophan or L-kynurenine leads to the production of KYNA but also to that of many other co-metabolites (including some such as 3-hydroxy-L-kynurenine and quinolinic acid that may be toxic). With the exception of chestnut honey, KYNA exists at relatively low levels in natural foodstuffs. However, its bioavailability is reasonable, and as the terminal element of an irreversible reaction of most tryptophan degradation pathways, it might be added exogenously without disturbing upstream metabolism significantly. Many examples, which we review, show that it has valuable bioactivity. Given the above, we review its potential utility as a nutraceutical, finding it significantly worthy of further study and development.

## 1. Introduction

Many natural products, including normal human metabolites, are of interest as candidate nutraceuticals since their deficiency, while not necessarily causing overt disease, may lead to a less-than-optimal functioning of the organism of interest [1]. Accordingly, improved functioning, and the potential for an extended and healthy lifespan, might then be realized by the addition of the nutraceutical. Research interest in such nutraceuticals, which when added to and delivered in food matrices are referred to as ‘functional foods’, is consequently considerable (e.g., [2,3,4,5,6,7,8,9,10,11,12,13,14,15,16,17,18,19,20,21,22,23,24,25,26,27,28]).

As part of a continuing survey of nutraceuticals, where we previously focused on ergothioneine (e.g., [18,29]), we determined that kynurenic acid (KYNA) might be of nutraceutical value. Here, we bring together some of the evidence that leads us to suppose that given its somewhat limited availability in normal diets, not least as the end product of a mammalian metabolic pathway, KYNA might indeed have a nutraceutical effect when provided exogenously.

## 2. Discovery, Structure and Some Biophysical Properties

**Figure 1:**
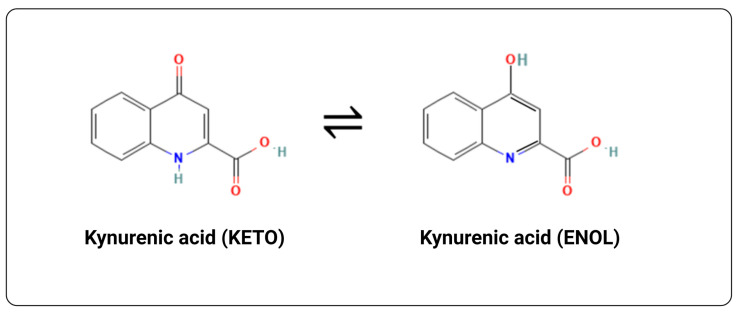
Kynurenic acid structure and tautomers.

Kynurenic acid (quinurenic acid, 4-oxo-1,4-dihydroquinoline-2-carboxylic acid, or 4-hydroxyquinoline-2-carboxylic acid) (https://pubchem.ncbi.nlm.nih.gov/substance/4854, accessed on 19 August 2024) was first identified in the urine of dogs by Justus von Liebig in 1853 [30]. It can adopt both keto and enol tautomers, as illustrated in Figure 1.

In aqueous solution at neutral pH, the keto form predominates [31,32,33], although a variety of crystal polymorphs are known [34]. It exhibits modest aqueous solubility (XlogP = 1.3) (pI~2.1 [35]), is very stable thermally [36,37], and can act as a photosensitizer [33,38,39] and metal chelator [40,41,42,43].

## 3. Biosynthesis and Phylogenetic Distribution

**Figure 2:**
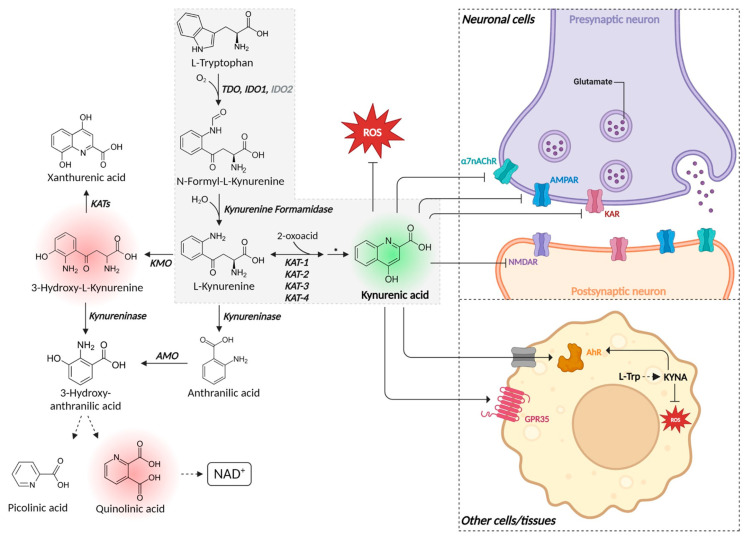
Elements of the kynurenine pathway. While the cytoprotective kynurenic acid can be derived endogenously from tryptophan, the pathway involves the synthesis of kynurenine that can lead to other toxic products such as quinolinic acid and 3-hydroxykynurenine. Redrawn in part from [102]. * Indicates ring cyclisation.

**Figure 3:**
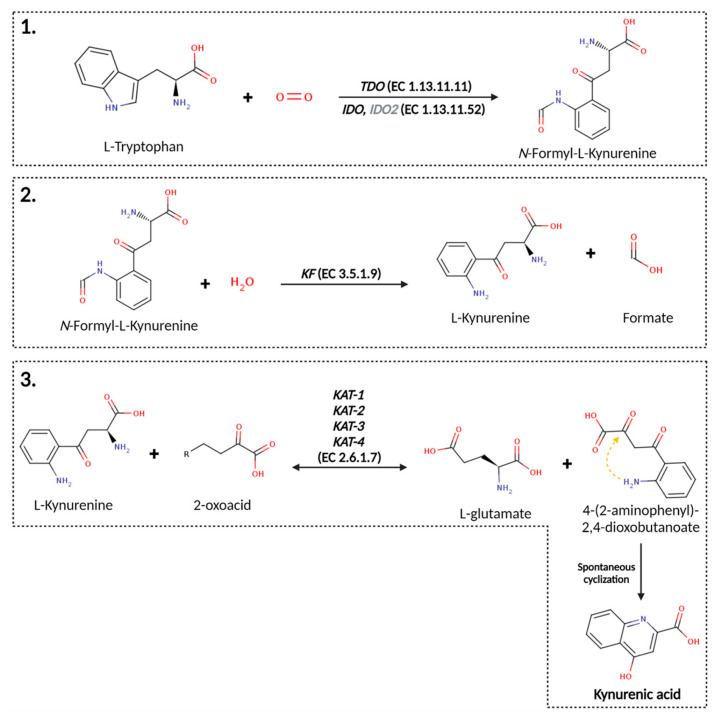
The ‘terminal’ steps of the KP from tryptophan to kynurenic acid. **1.** L-tryptophan is catalyzed to N-formyl-L-kynurenine (NFK) by tryptophan dioxygenase (TDO) or indole dioxygenase (IDO1, IDO2) (EC 1.13.11.11 and 1.13.11.52), depending on the organism/tissue. **2.** NFK is then converted to L-kynurenine (KYN) by the kynurenine formamidase (E.C. 3.5.1.9). **3.** Finally, KYN is catalyzed to the unstable 4-(2-aminophenyl)-2,4-dioxobutanoate intermediate by a kynurenine transaminase (KAT1-4) (E. C. 2.6.1.7), which is readily converted to KYNA by a spontaneous reaction. The spontaneous cyclization of the intermediate to KYNA is unique to KYNA biosynthesis, and it makes this reaction effectively irreversible meaning that exogenous KYNA will not be converted to L-kynurenine nor its toxic derivatives.

In humans, tryptophan is one of nine essential proteinogenic amino acids that must be obtained from the diet (i.e., we do not biosynthesize it [44]), while it (and its metabolites such as KYNA) may also be produced by the gut microbiome [45,46,47,48] (and transported in a manner reflected in the gut–brain axis [49,50,51,52,53,54,55,56,57,58,59,60]) or by some other minor pathways [61]. More than 95% of tryptophan is said to be degraded via the “L-kynurenine pathway” (KP) [62,63,64], consistent with mathematical models [65], and KYNA is one terminal part of the tryptophan degradation pathway, occurring via N-formylkynurenine and L-kynurenine (KYN) [66]. Note, however, that not all arms of this pathway exist in all cells: they are often segregated [67,68,69,70,71,72], implying the need for single-cell analyses [73,74,75,76,77,78,79]. While L-kynurenine is of interest as it is a precursor of NAD^+^ in eukaryotes (Figure 2) [80,81], our focus is on KYNA, made via the terminal stages of the pathway leading to KYNA from tryptophan (Figure 3), which goes via N-formyl kynurenine and L-kynurenine, and thus consists of three enzymes. Depending on the exact organism [82], these are tryptophan dioxygenase/indole dioxygenase (EC 1.13.11.11 and 1.13.11.52), kynurenine formamidase (E.C. 3.5.1.9) [83], and kynurenine oxoglutarate transaminase (E. C. 2.6.1.7). We note in passing that some members of the kynurenic acid pathway such as quinolinic acid [84,85] and 3-hydroxy-L-kynurenine [86] are considered neurotoxic (and KYNA can overcome this toxicity [87,88,89,90,91,92,93,94,95,96]) and so adding upstream elements, or elements that are more or less in equilibrium with them, is not necessarily a good idea. Additionally, KAT reactions in humans are normally considered to be of relatively minor significance due to the higher K_m_ for its two substrates (in the millimolar range), when compared with the K_m_ for L-kynurenine of the other two competitive reactions (catalyzed by kynurenine monooxygenase (KMO) and kynureninase A), that are in the micromolar range [97,98]. However (and see below), since KYNA is both seen as neuroprotective (e.g., [93,99,100]) and is essentially the terminal element and an irreversible step in this part of the kynurenine pathway, it is reasonable that provided it and its metabolites are beneficial or at least harmless, it can be added with impunity. Importantly, no diseases seem to be associated with the overexpression of KAT [101], the enzyme that is responsible for the synthesis of KYNA. The ability to add KYNA without affecting levels of molecules such as L-kynurenine directly is a core idea behind its potential use as a nutraceutical.

## 4. The Metabolic Pathway from Tryptophan to KYNA

Although a proper assessment of the flux to KYNA using metabolic control analysis (see [103,104,105,106,107]) seems not to have been performed, ODE-based mathematical modelling of the overall pathway has been [108], the interactions with vitamin B6 (pyridoxamine/pyridoxal) being of particular interest from a nutritional point of view as this is a cofactor for the KAT reaction. Readers are referred to the article [108] for a summary of the enzyme kinetic parameters of this pathway in mammals. Notably, both the K_m_ and k_cat_ values of the KAT enzyme(s) responsible for the production of KYNA from L-kynurenine are both rather high, indicating a tendency for linear increases in KYNA concentrations as that of L-kynurenine is raised [109].

Another ODE model for tryptophan degradation, notably in the liver, is given by Stavrum et al. [65], available at https://www.ebi.ac.uk/biomodels/MODEL1310160000 (accessed on 19 August 2024) and https://www.ebi.ac.uk/biomodels/BIOMD0000000602 (accessed on 19 August 2024), though the levels of KYNA are not reported in the paper. Importantly, the Copasi [110,111,112] files in SBML [113] version of the model available at https://www.ebi.ac.uk/biomodels/MODEL1310160000#Files (accessed on 19 August 2024) also indicate (i) that the KAT(1-4) reactions are seen as irreversible, and (ii) the flux-control coefficient (0.95) of the KAT steps on the flux towards KYNA is really dominating here. Of course, the values used may be varied, and a summary of kinetic parameters from different systems is given in Supplementary Information (Supplementary Table S1). Tissue distributions are tabulated in Supplementary Table S2.

Note too that a structural metabolic network model is also available as part of Recon2 [114] (https://www.ebi.ac.uk/biomodels/MODEL1311110001, accessed on 19 August 2024) and see [115]). Recon2 is a consensus network reconstruction based on the strategy used in [116] to produce one in baker’s yeast. Because just three enzymes catalyze the flux from the main dietary source of KYNA (viz L-tryptophan), we consider it worthwhile to review their properties in broad outline.

### 4.1. Tryptophan Dioxygenase/Indole Dioxygenase (EC 1.13.11.11 and 1.13.11.52)

Tryptophan dioxygenases (TDO) and indole dioxygenases (IDO) are heme-containing enzymes involved in the initial (and what is considered to be the most rate-limiting) step of the KP, catalyzing the oxidative cleavage of the indole ring of L-Trp to produce NFK (Figure 3) [117]. While IDO are widely distributed in the metazoan, in many bacterial species and fungi, and more recently have been identified in choanoflagellates and some ciliate species [118,119,120,121,122,123], TDO are also present in metazoan, bacteria and choanoflagellates, but have not been identified in fungi [124]. On the other hand, multiple IDO isoforms have been identified in fungal species [119,120]. Among them, IDOα isoforms usually show the lower K_m_ values while IDOβ isoforms show higher K_m_ but higher reaction velocities, resulting in higher catalytic efficiencies and suggesting that IDOβ could functionally substitute IDOα in fungal L-Trp metabolism for NAD^+^ production [120]. A third fungal IDO isoform, IDOγ, generally shows very low enzymatic activity for L-Trp (with catalytic efficiencies ranging around 1/100 of those determined for IDOα and IDOβ); however, IDOγ is very well conserved in fungi, suggesting that it might play an important role in those organisms, beyond NAD^+^ production [120].

Similarly, and apart from TDO, two distinct IDO genes have been identified in vertebrates. In humans, IDO1 and IDO2 are encoded by genes located in tandem on chromosome 8, which suggests gene duplication during evolution [125,126]. Although IDO and TDO catalyze the same reaction, there are fundamental differences between their structures, substrate specificity, tissue distribution and, consequently, function (see Supplementary Information).

In humans, IDO1 (hIDO1—UniProt P14902) has 403 amino acids, is monomeric, and with the exception of the liver, is widely distributed and constitutively expressed in multiple different tissues in healthy conditions, including lungs, small intestine, lymphatic system organs, female reproductive organs and the placenta [127,128]. Additionally, hIDO1 is constitutively expressed in several tumor cells, what is considered to make it a potential candidate for targeted anti-cancer therapy [129,130].

Under normal physiological conditions, IDO1 plays a fundamental role in immune regulation, acting as a checkpoint for the modulation of immune response mediated by antigen-presenting cells and exerting an immunosuppressive function, mediating maternal-fetal tolerance and protecting the fetus from maternal immune rejection [131]. In most cell types, however, hIDO1 is not only expressed constitutively under normal physiological conditions, but the enzyme is strongly induced in response to inflammation and infection stimuli, with IFN-γ being the main inducer [132,133], making IFN-γ-mediated hIDO1 induction and local L-Trp depletion an important factor able to inhibit pathogen growth [134].

In terms of enzyme activity, hIDO1 has the highest affinity for L-Trp (K_m_~20 μM) when compared with hIDO2 and hTDO, and it is also able to catalyze the oxygenation of D-Trp (K_cat_~2.97 s^−1^), although the K_m_ value for L-Trp is >100-fold higher than for D-Trp, suggesting a much weaker binding for D-Trp. Additionally, hIDO1 has a broad substrate specificity, catalyzing the oxygenation of a variety of indoleamines such as 5-hydroxytryptophan, 1-methyltryptophan, 5-methyltryptophan and 5-fluorotryptophan [135] and even serotonin in other organisms [136]. The activation of IDO1 activity requires a one-electron reduction of the heme—from the ferric (Fe^III^) state to the ferrous (Fe^II^) state—facilitating the binding of O_2_ and L-Trp to the ternary complex [135]. The superoxide anion radical (O_2_^●−^) is the reducing cofactor and co-substrate for the purified IDO1 enzyme [137].

The human homolog of hIDO1, hIDO2 (UniProt Q6ZQW0), is also a monomeric protein with 407 amino acids that displays enzymatic activity towards L-Trp, although with a much higher K_m_, around 6.8 mM, when compared with hIDO1 and hTDO [138,139]. This value is more than 100-fold higher than typical physiological L-Trp levels [140,141], making it questionable if IDO2 plays a direct role in L-Trp metabolism. Yuasa and Ball showed that hIDO2 expression in *Saccharomyces cerevisiae* strains auxotrophic for nicotinic acid was not able to rescue the auxotrophic phenotype in the yeast, while expression of hIDO1 was, suggesting that the lower activity of hIDO2 might not be enough for NAD+ synthesis in yeast [121].

hIDO2 is not well characterized and little is known about its tissue distribution—at least at the protein level—and function. That is mainly due to (i) the complexity of hIDO2 transcription and, (ii) the lack of an accurately validated antibody. The hIDO2 gene generates five alternative transcripts, of which only one encodes the full-length protein [126]. Additionally, the gene contains two functional polymorphisms in the coding sequence: the first one, a nonsynonymous substitution (R248W) reduces hIDO2 catalytic activity by ~90% and the second one, a premature stop codon (Y359X), completely abolishes it. These polymorphisms have high prevalence in some populations—up to 50% [126].

Full length hIDO2 mRNA was detected in placenta and brain by RT-PCR, while primers specific for the hIDO2 exon 10 (common to all hIDO2 transcript forms) detected hIDO2 mRNAs in the liver, intestine, thymus, lung, spleen and kidney [126]. At the protein level, a few studies have identified hIDO2 in lungs, dendritic cells and in the interface between the placenta and the fetus [142,143,144].

By contrast to hIDO2, mouse IDO2 (mIDO2) is better studied. The constitutive expression of mIDO2 was detected in many organs at the protein and mRNA level (see Supplementary Table S1) and additionally, mIDO2 mRNA was upregulated in dendritic cells and mesenchymal stem cells treated with IFN-γ [132]. A recent study showed that IDO2 mediates autoreactive B-cell responses in mice, contributing to an exaggerated inflammatory response and the severity of joint inflammation in a model of autoimmune arthritis [145].

Unlike IDO1 and IDO2, hTDO (UniProt P48775) is a tetrameric enzyme [146] that is primarily confined in the liver and brain, where it seems to remain unresponsive to immunological stimuli; therefore, functioning as the main regulator of systemic tryptophan levels under physiological conditions [147]. As the main ‘housekeeping’ (see [148]) enzyme responsible for metabolizing the dietary tryptophan, hTDO is upregulated when the blood concentration of tryptophan rises above ‘physiological’ levels [149]. hTDO is well studied as a potential drug target, as its mRNA expression appears to be upregulated in many tumor types [150]. As it is mainly expressed in the liver and brain, human hepatocarcinoma usually present increased hTDO expression and many studies have suggested the involvement of hTDO in CNS diseases such as Alzheimer’s, Parkinson’s and Huntington’s disease [151,152,153].

Another difference of hTDO when compared to hIDO is that hTDO has a high substrate specificity, L-Trp being the only relevant physiological substrate, although there is evidence for oxidation of D-Trp, but with a very low activity, (low k_cat_ at the high concentration of D-Trp tested) [154]. Additionally, the binding affinity of L-Trp to the ferric and ferrous forms of hTDO is very similar, suggesting that hTDO does not specifically favor substrate binding to the ferrous enzyme, as observed for hIDO [154].

### 4.2. Kynurenine Formamidase (E.C. 3.5.1.9) [83]

Human kynurenine formamidase (KF) (UniProt Q63HM1) or arylformamidase (Afmid) has 303 amino acids and catalyzes the second step of the KP from L-trp to KYNA by converting NFK to KYN (Figure 3). Its Alphafold-calculated structure is available, although very little is known about the human KF (hKF).

Pabarcus and Casida predicted [83] and later identified [155] the catalytic triad of the mouse KF (mKF). They showed that point and combined mutations in the Ser162, Asp247, and His279 (S164, D147 and H279 in the human protein—our unpublished alignment using the tools provided in [156]) completely abolished the conversion of NFK to KYN [155].

In mice, mKF is primarily expressed in the liver and to a less extent in the kidney [157]. *Afmid* knockout mice showed elevated plasma concentrations of NFK, KYN and KYNA (as well as kidney failure), consistent with low levels of mKF expression [158]. In *S. cerevisiae*, a KF knockout strain showed an accumulation of NFK and a slow growth phenotype in absence of exogenous nicotinate [159]. A range of compounds, including organophosphate and methylcarbamate insecticides are potent inhibitors of KF [160,161], and in vivo treatment of mouse with organophosphorus acid triester diazinon resulted in the accumulation of NFK and reduced KYN biosynthesis in the liver and a 5-fold increased plasma KYN and 5- to 15-fold increased concentrations of KYNA and xanthurenic acid in urine [162]. This suggested a strategy for the development of safer insecticides of this type [163].

### 4.3. Kynurenine Oxoglutarate Transaminase (E. C. 2.6.1.7)/Kynurenine Aminotransferase (KATs)

Overall, the simple pathway structure alone, plus other observable properties such as the modelling above and correlations between KAT levels and KYNA concentrations, leads one to suppose that this reaction, as catalyzed by various KATs (kynurenine aminotransferase), is especially important to the synthesis of KYNA. The reaction (BRENDA, https://www.brenda-enzymes.org/enzyme.php?ecno=2.6.1.7, accessed on 19 August 2024) has been shown to be effectively irreversible [164,165] in the direction of KYNA synthesis, and is treated as such in the ODE models [65]. The transamination of kynurenine by KATs yields an unstable keto acid product, 4-(2-aminophenyl)-2,4-dioxobutanoate, which is spontaneously and rapidly cyclized to KYNA [82,166] (Figure 3). This, importantly, is what makes this step functionally irreversible. KAT orthologous are found in all kingdoms [166], and a cross-species comparison of KAT structures shows a high conservation of the monomer architecture, consisting of an N-terminus arm, a small and a large domain [66,167,168].

Depending on the microbe, the function of KYNA is unclear, as inhibiting its significant production by inhibiting the relevant (Aro8/9) KAT enzymes in yeast, for example, has no significant effects [169]; arguably this enzyme activity may help detoxify excess tryptophan [169] (and excess amino acids can certainly be toxic to microbes [170]). By contrast, Genestet et al. observed that *Pseudomonas aeruginosa* clinical isolates present a high transcription level of the *kynaA* gene (the first gene involved in the kynurenine pathway, converting L-Trp to L-kynurenine) and produce high amounts of kynurenine when in contact with human neutrophils, leading to increased bacterial survival. By testing kynurenine-overproducing (Δ*kynU*—gene involved in the conversion of L-kynurenine into L-anthranilate) and kynurenine-deficient (Δ*kynA*) *P. aeruginosa*, they determined that L-kynurenine inhibits ROS production by neutrophils, but when testing the specific mechanisms, they failed in showing that L-kynurenine had a direct effect on NADPH oxidase (the main ROS producer in neutrophils) or was a potent scavenger of superoxide anions in a superoxide-producing nonenzymatic PMS–NADH system. In contrast, KYNA appeared as the best scavenger of O_2_^−^ among the molecules tested [171]. In this case, an increase in L-kynurenine production by *P. aeruginosa*, especially when the pathway branch leading to anthranilate production is suppressed, might lead to an increase in the production of KYNA [172], which appears to be the best scavenger, although more studies are necessary to define its role over L-kynurenine in *P. aeruginosa* survival. Furthermore, KATs can play an essential role in bacterial survival and amino acid synthesis/nitrogen assimilation due to their higher affinity and efficiency for other natural substrates over L-kynurenine [168,173].

There are four human KATs, summarized by Rossi [66] and structures are available, e.g., for KAT1 [174,175], KAT II [176,177,178,179], KAT III (as a homology model [180]) and KAT IV [181]. There are certain differences in substrate specificity, but all are homodimeric pyridoxal phosphate-dependent enzymes [165]. Because of their importance to endogenous KYNA synthesis, we consider each in turn.

### 4.4. KAT1

The main isoform of KAT1 (Uniprot Q16773) has 422 amino acids, a broad substrate specificity as an aminotransferase and also catalyzes β-lyase reactions using several cysteine *S*-conjugates as substrates. It exhibits a preference for glutamine as amino donor (see Supplementary Table S2) and was demonstrated to have aminotransferase activity towards 5-*S*-L-cysteinyldopamine, the cysteine S-conjugate of dopamine; this is significant as 5-S-L-cysteinyldopamine is neurotoxic and markedly increased in the substantia nigra of patients who died of Parkinson’s disease [182,183]. As judged by the protein atlas [184], KAT I is widely distributed in human tissue, including brain [185].

In vitro KAT-1 can use many α-keto acids as amino group acceptors and although it has detectable activity on oxaloacetate and pyruvate, the specific activity on these two α-keto acids is very slow, making unlikely that they are physiological substrates for human KAT I [186]. Additionally, hKAT1 conversion of L-kynurenine to KYNA (using α-ketobutyrate as α-ketoacid) is strongly inhibited by tryptophan, phenylalanine, glutamine and cysteine (2 mM of each amino acid inhibits over 50% of aminotransferase activity) and by indo-3-pyruvate (0.2 mM inhibits 50%) [186]. In vivo preference of L-glutamine over L-kynurenine in brain is discussed by Cooper et al. based on L-glutamine and L-kynurenine availability/concentrations and their K_m_ and k_cat_ for KAT I, suggesting that the capacity of KAT I to utilize L-glutamine is many orders of magnitude higher than the capacity of the enzyme to utilize L-kynurenine [187]. This makes it very unlikely that KAT-1 has a major role in KYNA production in the brain. Indeed, many studies have shown that KAT II is the main enzyme responsible for KYNA production in the brain; however, KAT II knockout in mice led to a reduction of 71% in the KYNA levels in the brain [188]. We note that Kapoor et al. [189] showed that the enzymatic activity of KAT I in the brain of patients with schizophrenia was altered, and suggested that the enzyme might play an important role in KYNA synthesis in the brain; however, those two facts do not follow, and the observation does not seem to have been followed up.

### 4.5. KAT II

KATII (Uniprot Q8N5Z0), 425 residues, has a very broad substrate specificity, albeit with a preference for glutamate L-kynurenine, a K_m_ for kynureine of 4.7 mM and a k_cat_ of 585 ·min^−1^ (ca 10 ·s^−1^) while a catalytic efficiency of 196.2 mM^−1^·min^−1^ for aminoadipate is reported [177]. Its mechanism of action is known in detail [165], its molecular dynamics simulations are given by [190], and its pharmaceutical inhibitors are summarized, e.g., by [178,191,192,193,194,195]. KAT II is considered the major source of KYNA in mammals, and is widely distributed, not least in the liver. The interest in developing pharmacological inhibitors comes from the somewhat variable data (Table 4, below) indicating that KYNA levels can sometimes be raised in various kinds of bipolar disorder [196]. From the perspective of this review, however, we think it more likely that any beneficial effects of such inhibition may be mediated via other parts of the tryptophan degradation pathway that are likely to change simultaneously (see Figure 3, and, e.g., [197,198]), and indeed we are not aware of any marketed drug for such disorders based on selective inhibition of KAT II enzymes.

Unlike KAT I, the conversion of L-kynurenine to KYNA by KAT II is not significantly inhibited by other amino acids [177]. Taken together, preference for aminoadipate and L-kynurenine and the lack of inhibition by other amino acids might explain the importance of KAT II in preventing neurotoxicity. KAT II is responsible for as much as 75% of KYNA synthesis in most brain areas [61,199] and its downregulation has been related to numerous brain diseases involving KYNA depletion [200]. KATII might also be involved in the regulation of brain levels of aminoadipate [201], a toxic metabolite for astrocytes in vitro and in vivo and part of lysine metabolism in the liver [201,202,203].

Regarding sequence and structure, KAT II has a predicted 22 N-terminus mitochondrial signal sequence, targeting the enzyme to the inner membrane of mitochondria [204]. KAT II enzymes do not belong to any of the previously existing fold type I aminotransferase groups from the α-family of PLP-dependent enzymes, but to a new subgroup called Iε. This occurs because hKAT II have a highly flexible N-terminal fraction, residues 15–33, that is able to move far from and closer to the active site upon substrate binding, thus accommodating different substrate sizes, which can explain its broad substrate specificity [177]. The swapping of the catalytic N-terminal region is unique in this subgroup of aminotransferases [109]. The conformation adopted by the N-terminal region in human KAT II is similar to the one observed in the N-terminus of members of PLP-dependent lyases [66], and in fact, hKAT II shows β-lyase activity towards various cysteine S-conjugates and β-chloro-D,L-alanine [205].

Both hKAT I and hKAT II are by far the most well studied human KATs. Both enzymes have been largely targeted in structure-based drug design aiming (we think unadvisedly) at the lowering of KYNA levels. Among the most potent KAT I inhibitors are the phenylhydrazone hexanoic acid derivatives and, for KAT II, the pyrazole compounds. The inhibitors showed effectiveness in reducing KYNA production followed by an improvement in alleviating cognitive dysfunction in animal models, but studies in humans are yet to be performed [206,207], and mechanisms are far from clear cut.

### 4.6. KAT III

KAT III (Uniprot Q6YP21), most closely related in sequence to KAT I (unpublished alignment search using the tools provided in [156]), has 454 amino acids and, like KAT I, a preference for glutamine as amino donor. Like KAT I, it is more or less ubiquitously distributed in humans and shares a similar intron-exon organization, except for the presence of exon 2, which encodes a 33-amino acid sequence that corresponds to the leader sequence for mitochondrial targeting. Exon 2 can be alternatively spliced in hKAT III, and thus the enzyme can be found in the cytoplasm or mitochondria [204,208]. The human KAT III has not been biochemically characterized, but the mouse KAT III (mKAT III), which shares 86.8% similarity and 83.7% identity with the hKAT III, has been fully characterized. Here, we discuss the biochemical/kinetic parameters of the mKAT III. Similarly to hKAT I, mKAT III has a preference for glutamine as amino donor. The catalytic efficiency for L-kynurenine is 92 min^−1^ mM^−1^ and the transamination of L-kynurenine to form KYNA is significantly inhibited by methionine, histidine and glutamine (~75%), leucine and cysteine (~50%) and phenylalanine (~25% inhibition) [209].

### 4.7. KAT IV

Better known as the mitochondrial glutamate-oxaloacetate aminotransferase 2 (GOT2), KAT IV (Uniprot P00505) has 430 amino acids and catalyzes the reaction of 2-oxoglutarate and L-aspartate to L-glutamate and oxaloacetate, playing an essential role in the malate-aspartate shuttle in mitochondria and in the synthesis of glutamate [210]. It is very widely distributed in human tissues, and an experimental [181] and Alphafold-calculated structure is available. KAT IV has been shown to play significant role on KYNA synthesis in human and murine brains [211]. Biochemical characterization of mouse mitochondrial KAT IV (mKAT IV) showed high transamination activity towards glutamate and aspartate as amino donors and lower but detectable activity towards phenylalanine, tyrosine, cysteine, tryptophan, 3-HK, methionine, kynurenine, and asparagine [109]. As amino group acceptors, mKAT IV showed similar K_m_ values for phenylpyruvate and oxaloacetate, but higher catalytic efficiency towards phenyl-pyruvate (58 mM^−1^·min^−1^) [212]. Accordingly, the transamination of L-kynurenine to KYNA was significantly inhibited by glutamate and aspartate, and in lower amounts by cysteine, glutamine, phenylalanine, tryptophan and tyrosine [109]. Notably, KAT IV was the one that showed substantially increased activity following endurance exercise (as did KYNA) [213,214,215].

The overall conclusion here is that most tissues exhibit some basal KAT activity, consistent with the view that KYNA is a useful metabolite for mammals.

## 5. Transport of Kynurenic Acid and Related Metabolites

### Mammalian kynurenic Acid Transporters SLC22A6 and SLC22A8

**Figure 4:**
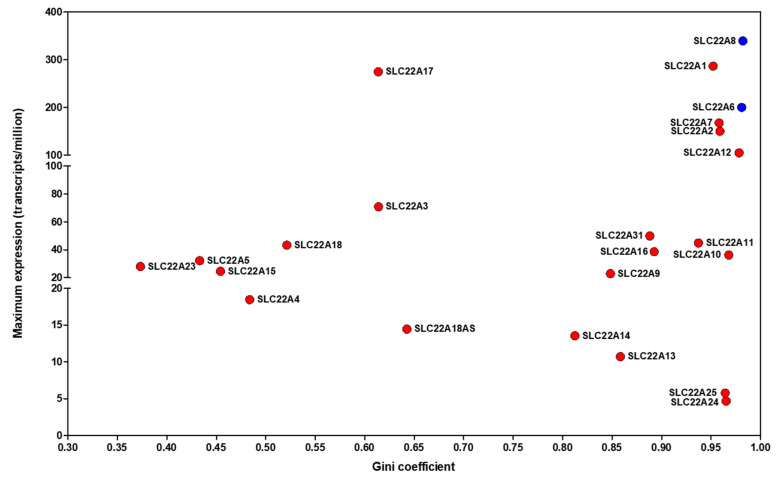
Transcript expression levels and Gini coefficient of SLC22A6 and SLC22A8. Data from [148,238].

As is now well established, molecules such as KYNA require protein transporters to cross cell membranes [216,217,218,219,220,221,222,223,224,225,226,227,228]. Human transporters are classified into two superfamilies. The SLCs, for SoLute Carriers [229,230], are either equilibrative (effecting ‘facilitated diffusion’) or use ion electrochemical gradient to transport their substrates against ostensible concentration gradients (‘concentrative’). In addition, there are various ATP-binding cassette (ABC) transporter families, commonly encoding efflux transporters [231]. However, exceptions exist, and some are actually influx transporters [232,233,234]. The SLCs described to date as being involved in the transport of kynurenic acid are the related SLC22A6 and SLC22A8, which come from the ‘organic anion transport’ or OAT family [235,236] and were previously known as OAT1 and OAT3. They are polyspecific transporters with a very wide substrate range among anions [237], but are Na^+^-independent and not thought to be concentrative (unless balanced by an opposite starting concentration gradient of another substrate). Moreover, using the tissue data from [148,238], while their maximum expression profile levels are quite respectable among SLC22 family members (Figure 4), these gene products have an exceptionally high Gini coefficient (see [148,239]). This means that they are mainly expressed in a very small number of tissues (in this case the kidney, urinary bladder, and (for SLC22A8) brain (see e.g., https://www.proteinatlas.org/ENSG00000149452-SLC22A8/tissue, accessed on 19 August 2024), and not, for instance, the liver. This said, while it is stated that the blood–brain barrier itself is poorly permeable to KYNA [89], the original paper [240] on which the statement is based indicates that its rate of uptake is only ~10-fold lower than that of L-kynurenine (which, unlike KYNA, is a substrate of the LAT1/SLC7A5 transporter [241,242]). Correspondingly, we would argue that KYNA can in fact enter the brain if supplied exogenously, albeit the transporters (of which there may be many [243]) are as yet unknown. Seemingly, KYNA (and its 7-chloro derivative) can also be effluxed by MRCP4 (ABCC4) as well as by SLC22A6/8 [242,244], as each of these transporters is inhibited by probenecid, whose presence led to an 885-fold increase in the concentration of 7-Cl-KYNA in the prefrontal cortex of rats [242] (see also [240,245,246,247,248,249,250]). This may also help to account for the low apparent net uptake sometimes seen, as ABCC4 is a very efficient efflux pump [251], and efflux pumps in general necessarily tend to have more influence on steady-state levels of a drug than do influx transporters [252]. Indeed, they are a major cause of resistance to antibiotics (e.g., [232,253,254,255]) and to antitumor drugs (e.g., [256,257,258,259,260]). Therefore, the tissue distribution of KYNA is likely to depend strongly on the concentrations of potential ABCC4 inhibitors (some listed in Table 1) as well as the disposition of efflux transporters like ABCC4 and others of the ABCC family. Interestingly, among the most potent inhibitors of ABCC4 (K_i_~1 μM [251]) is the flavonoid quercetin, another important nutraceutical [261,262]. ABCG2 (BCRP) is also a potential effluxer of KYNA [263,264]. Note too that kynurenic acid may also be bound to albumin [265,266], something that would be missed in standard extractive metabolomics studies. Overall, the system is extremely complex, benefitting strongly from the kind of ODE-based modelling that is known in this field as ‘physiologically based pharmacokinetic modelling’ [267,268], while the increasing availability of cell lines engineered to overexpress individual SLCs [269] should make answering this question of KYNA transporters much more accessible [270].

**Figure 5:**
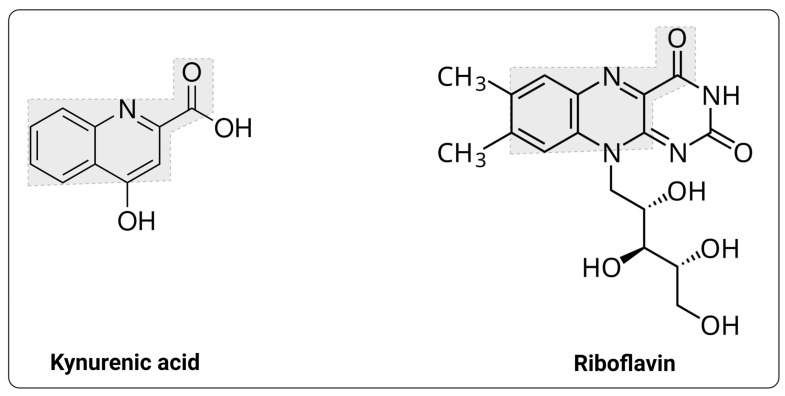
2D chemical structures of kynurenic acid and riboflavin, indicating a common substructure.

This probably means that, given also the much higher concentrations of KYNA in tissues versus plasma (see below), there are other kynurenic acid transporters waiting to be discovered. In this context, interestingly, a kynurenine monooxygenase inhibitor was transported via the riboflavin transporter SLC52A2 [85], and the structures of KYNA and riboflavin are given in Figure 5. There are also some weak indications [288] that kynurenates might be substrates of SLC1 [289,290] family members. Other obvious candidates are members of the SLC16 monocarboxylate transporter family [291,292,293]. KYNA transporters in other organisms are surprisingly poorly characterized. An especially striking finding [294] was that KYNA was accumulated 20-fold in cord blood relative to the maternal plasma, and its concentration was also 20-fold greater (in mice) in the fetal brain vs. maternal tissue [295] (see also [296] for external KYNA addition), strongly implying a role for both concentrative transporters and for KYNA itself in fetal development.

**Figure 6:**
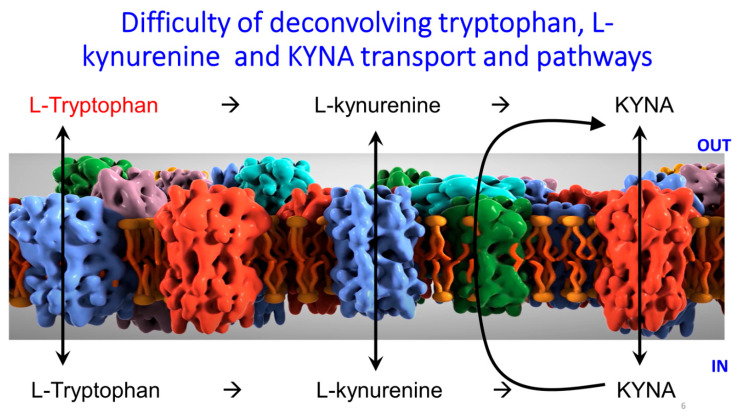
Assessing the fate of KYNA when its precursors are added externally is fraught unless one knows the expression levels of all the relevant transporters, the direction in which they transport, and whether they are concentrative or equilibrative [223,228], and we only know the existence of some of them. The membrane is redrawn in part from the animation at https://www.youtube.com/watch?v=s23vNwLE-Jw, accessed on 19 August 2024.

We note also that L-kynureine is more widely bioavailable, being a substrate of the ‘large amino acid transporter’ in *C. elegans* [297], a homologue of the human SLC7A5 [241,298,299,300,301,302] that transports many large, neutral amino acids, including tryptophan and L-kynurenine). This of course complicates analyses of the transport of KYNA if tryptophan or L-kynurenine and/or kynurenine amino-transferase(s) are also present or added (Figure 6). D-kynurenine can also be used and is metabolized via D-amino acid oxidase [303] or transamination [304].

## 6. Bioavailability

Given the relative paucity and activity of (known) transporters, it is possible that KYNA is not the most bioavailable of nutraceuticals, but that oral KYNA is definitely absorbed in the mammalian gut [36,305,306,307,308], and can cross a model of the blood–brain barrier with a respectable permeability of some 3.5·10^−6^ cm·s^−1^ [309], precisely that of the modal value for marketed drugs across Caco-2 cells [310]. The levels of natural absorption can also be improved further by linking it to a transporter substrate [311,312] or using special formulations [309,313,314,315,316,317,318] (we ignore analogues that do not yield KYNA itself, since our focus is the nutraceutical activity of the genuine natural product). Thus, there is no reason why exogenously supplied KYNA might not be bioavailable, and the many effects reviewed here surely indicate both that it is and that can confer host benefits.

## 7. Concentrations of KYNA in ‘Normal’ Serum and Plasma, and Other Body Fluids/Tissues

Concentrations in the plasma of the nutraceutical ergothioneine are typically 1–4 μM (~229–916 ng/mL) (e.g., [319,320]), and are ~10-fold higher in whole blood as ergothioneine is concentrated in erythrocytes [18]. In contrast, KYNA concentrations in plasma and serum are some 1–2 orders of magnitude lower (Table 2). Moreover, the KYNA plasma concentration is normally far lower than the ~5 μM K_m_ values measured [321,322] for SLC22A6/8 (and indeed for many of its putative receptors—see [323] and below). The median levels are fairly consistently ~30–50 nM in plasma or serum across a very wide range of studies (Table 2). This relative constancy also implies a significant degree of regulation [324]. With a MW of 189 at pH 7, 50 nM equates to some 9.45 ng KYNA·mL^−1^ (one paper gives values of KYNA in children that are orders of magnitude different [325], and another [326] gives very unusually low values; these are not included in Table 2). A recent meta-analysis showing a tendency of serum/plasma KYNA to increase with age is given by [327], with similar data in [328,329], though tissue changes are rather variable [330].

### 7.1. Breast Milk

O’Rourke et al. measured various tryptophan metabolites including KYNA in human breast milk, finding values of ca. 12 ng/mL (52 nM) at term, rising ~3.5-fold for term babies, but less so (and not significantly) in pre-term babies. Milart et al. [359] noted that natural human breast milk contained KYNA at much higher levels (from ca. 21 nM to ~300 nM at 6 months of breastfeeding, a ca. 14-fold increase) as lactation kicked in, levels that were well above those in a variety of commercial infant formulas. Rat pups exposed postnatally to KYNA also demonstrated less obesity for the same increase in bone mineral density [359] (see also [360,361]).

### 7.2. Bile

Concentrations of KYNA in bile are well in excess of those in plasma/serum, with values of >800 nM [308,362] being reported in humans. 

### 7.3. Intestine

Of course, depending on dietary sources of tryptophan and kynurenic acid, plus the variable ability of microbes in the gut to metabolize these molecules to KYNA, mean that KYNA levels could in some cases be quite high, and this has been reported (e.g., [334]).

### 7.4. Gut Microbiota and KYNA

The gut microbiota composition plays an important role in regulating KP metabolites, which subsequently influence host immune response. The interplay between these three is tightly controlled and complex; and the gut microbiota can influence health and disease through fine-tuning KP metabolites [50,52,363].

In the gastrointestinal (GI) tract, the function of AhR signalling as a critical regulator of gut immune function has been extensively reported [364,365,366,367,368,369]. Therefore, KYNA—as well as other members of the KP—may be an important mediator in this complex crosstalk, once it acts as a direct ligand of the above-mentioned receptor, activating it locally and systemically [51,370]. In fact, the absence of AhR causes an increase in endogenous KYNA levels in mice [93] and, in a recent study, gut microbiota-derived KYNA and other metabolites from tryptophan metabolism were shown to be the main AhR activators in the GI tract [371].

Another important role of ligand-activated AhR is the induction of IDO1 activity through activation of pro-inflammatory cytokines [372]. In this case, it is important to consider the influence of altered IDO activity on KP metabolite production [373].

In addition to AhR, transmembrane G protein-coupled receptors (GPCRs) also play an important role in GI tract homeostasis and intestinal immunity [374,375,376]. Among the GPCRs, GPR35 is predominantly expressed in the GI tract and, since KYNA is a known GPR35 ligand, several studies have suggested that KYNA acts as the link between gut-microbiota homeostasis and host immunological regulation. For example, Wang and collaborators demonstrated that GPR35 activation by KYNA is a necessary component to maintain gut homeostasis, regulating the progression and outcome of colitis in an ulcerative colitis-induced rat model [377]. Another study demonstrated that KYNA-mediated AhR and GPR35 regulation maintain intestinal integrity and homeostasis in a chemotherapeutics-induced intestinal damage model. Sensitivity differences of AhR and GRP35 to KYNA leads to a primary accumulation of KYNA through AhR-IDO1 positive feedback regulation. Accumulation of KYNA then is sensed by GPR35, which ameliorates intestinal injury and restores gut homeostasis [378]. Additionally, Miyamoto and collaborators have demonstrated [379] that the increased KYNA levels in the small intestine mediated by the microbiota modulates the recruitment and aggregation of GPR35-positive macrophages, ultimately triggering the onset of experimental autoimmune encephalomyelitis.
**Tissues—Rodents**

**Concentration Range****Comments****Reference**
Review[347]32 nM increases to → 135 mM after dosingGerbil brain [360]1–16 mMRat ileum[361]~40 nM in plasmaTrebled after dosing at 5 mg/kg[210]**Tissues—human**

**Concentration range****Comments****Reference**
Review[305]0.2–0.7 pmol/mgBrain; 3× increase in Down syndrome[362]2–3 pmol/mgBrain[353]Up to 1.58 pmol/mgBrain[363] and review [347]1.6 mMColon[359]10.2 ng/mLFetal membrane[364]7.6 g/mLUmbilical Cord[364]1 ng/mLPlacenta[364]

If we loosely assume a unit density (1 g·mL^−1^) for tissue, 1 pmol.mg^−1^ equates to 1 μM, considerably higher than serum/plasma levels, strongly implying that there is concentrative uptake driven via one or more (presumably H^+^- or Na^+^-coupled) transporters, whose identities—as with that of many SLCs [269]—remain unknown. Although it was assumed that it was the local rates of production that varied, this conclusion is also consistent with the analyses of maternal, fetal and cord blood in [380].

### 7.5. Urine

As reviewed by Turska et al. [308], urine concentrations of KYNA tend to be in the micromolar range, from ca. 4 μM [381] to more than 20 μM [382]. It is hard to know how much of this is due to simple synthesis from L-kynurenine in the kidney (for which there is no particular reason) and how much is due to concentrative efflux (noting again that SLC22A6/8 are considered to be exchangers [383,384] and not concentrative. Note that while SLC22A6 (OAT1) https://www.proteinatlas.org/ENSG00000197901-SLC22A6/tissue, accessed on 19 August 2024 and SLC22A8 (OAT3) https://www.proteinatlas.org/ENSG00000197901-SLC22A8/tissue, accessed on 19 August 2024 are highly expressed in the kidney, ABCC4 is not expressed in the kidney https://www.proteinatlas.org/ENSG00000125257-ABCC4/tissue, accessed on 19 August 2024.

### 7.6. Feces

These are somewhat infrequently measured, but in one rat study [385] levels were around 100 ng/g (~0.5 μM if 1 g ≡ 1 mL), rising to 40 times that in the presence of a kynurenine monooxygenase inhibitor.

## 8. Nutritional Sources

As a metabolite of an essential amino acid, KYNA is widely distributed, and plants can take it up from the soil [386]. The literature for natural products is focused on *Ephedra* spp. (e.g., [387,388]), which have a significant use in traditional Chinese medicine (MaHuang), but the contribution to this of KYNA is unknown and many *Ephedra* alkaloids can be toxic. Besides culinary herbs [55], where the richest sources are basil and thyme [307], or medicinal herbs that still might provide at most a few tens of μg [386,389], of those vegetables consumed in reasonable quantities, broccoli and potatoes seem to have the highest values (Table 3). As with many amino acids [390], the levels varied massively between different cultivars [391], warranting more detailed studies.

KYNA contents were also measured in tea and coffee, but did not exceed 8.7 and 0.63 μg/100 mL, respectively.

**Figure 7:**
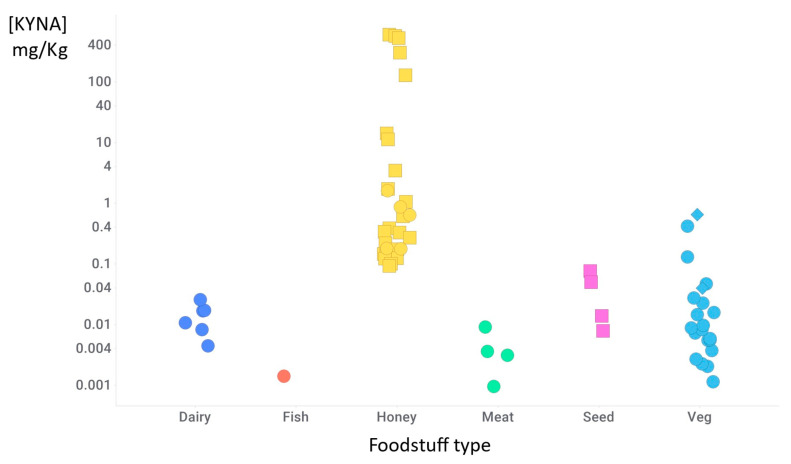
A summary of the kynurenic acid concentrations of various foodstuffs. Data are compiled from the following references: squares [392], circles [36], diamonds [391].

Honey is a notable source of KYNA. In particular, the product from sweet chestnut trees reportedly weighs in at ca. 100 mg KYNA/kg [36,307] or even more [392] (Table 3 and Figure 7). The source of these high levels is, in particular, the male flowers of the tree, most other parts of the edible chestnut having far lower levels [392]. Note, however, that most honeys are closer to 1 mg/kg or lower, so a 25 mg supplement (say) of KYNA would require a mighty dose of any but the most potent honey. An overall conclusion from Table 3 is that if KYNA is going to be given as a nutraceutical, even at low doses, its levels are likely to exceed those seen when its sole exogenous source is foodstuffs [308] (propolis, of an unstated origin, was also said to be a good source [36], although KYNA was not reported in a number of untargeted metabolomics studies [394,395,396,397], so this seems worth investigating further).

As with ergothioneine, where clear (even striking) benefits are seen from eating mushrooms that contain it, e.g., in preventing mild cognitive impairment [398], even though they may contain nutraceuticals beyond the one of focus, chestnut honey is clearly the equivalent for KYNA. Thus, while the precise contribution of KYNA itself is unknown (chestnut honey also contains many phenolic and other antioxidants [399]), the excellent review by Turska and colleagues [308] does provide a list of examples where this honey is thought to have provided health benefits. These include positive effects on glucose metabolism and neurodegeneration in obese mice [400], vs. high-fat diets in obese mice [401], acid-/alcohol-induced gastric ulceration in mice [402], on carbon tetrachloride-induced liver damage in rats [403], and in inhibiting breast cancer cell line proliferation in vitro [404]. It has been found protective against influenza in mouse macrophages and mice in vivo [405]. Along with curcumin, it also produced a substantial increase in the longevity of heat-stressed bees [406].

## 9. Pharmacokinetics

There have been few studies of the pharmacokinetics of exogenous KYNA [308], with Turska’s study in mice [407] being the stand out. Here, radiolabelled KYNA was provided intragastrally (calculated as ~5 nmol at 20 μM), and its appearance in blood, liver and spleen noted, indicating uptake into these organs. Kidney levels were not reported. Most of the KYNA was excreted in urine in under 24 h, while liver retained a significant level of radioactivity at that time. Note that liver in humans does not express the two known transporters (see above), nor significant amounts of the ABCC4 effluxer, implying the need for other, as yet unknown, transporters.

## 10. Further Metabolism and Excretion

In humans, KYNA is largely seen as a terminal step of tryptophan degradation [308], and as noted above is excreted in urine via the kidneys. As such, metabolic transformations are not considered a major feature of KYNA ingestions, though Takahashi et al. reported some conversion to quinaldic acid [305], the dehydroxylated variant of KYNA [408]. Various bacteria can of course metabolize it, e.g., certain pseudomonads can assimilate and metabolize it to glutamate, alanine and various organic acids [409], but they can also excrete it [172].

## 11. Oxidative Stress

Oxidative stress is extremely widespread in a whole host of chronic, inflammatory diseases [410,411], so much so that there were over 125 papers having the terms “oxidative stress” and “review” in their titles alone at Web of Knowledge just for 2023. Since we have reviewed elements of it three times recently [410,412,413], we do not repeat this further here, save to mention that the chief cause is the production of ‘reactive oxygen species’ (ROS) such as peroxide, superoxide, and—as catalysed by free iron [324,410,414,415]—the especially nasty hydroxyl radical OH^•^. Any small antioxidant molecules that can react with such ROS (also known as ROS scavengers) are thus likely to ameliorate oxidative stress, and KYNA certainly has this property [416,417,418,419,420] (and see below). Below we discuss other mechanisms that may account for the ability of low concentrations of KYNA to help deal with oxidative stress.

## 12. Diseases in Which KYNA Levels Are Significantly Altered

Table 4 summarizes some of the diseases or syndromes in which normal levels of KYNA are raised (occasionally) or (more frequently [421]) lowered. In the former cases, there is some evidence that this is actually the host’s homoeostatic attempt to combat the causes of the disease. Note, of course, that in many cases it is probable that it is an increase in tryptophan and its degradation pathway metabolites more generally that are changed, so increases in levels upstream may themselves correlate with KYNA levels yet themselves be responsible for physiological or biochemical effects [422]. The importance of the kidney as the main means of excretion (via urine) is highlighted by the very high levels of plasma KYNA reached in various kidney diseases.

## 13. KYNA and COVID-19

The arrival of the COVID-19 epidemic caused by the SARS-CoV-2 virus has had profound effects on the world, as well as on scientific approaches to the understanding of both acute diseases and their post-acute or chronic sequelae (‘Long COVID’) [486,487]. A number of studies have highlighted changes in tryptophan metabolism and in KYNA production in particular as a response to the virus. Thus, Thomas et al. [488] found trp metabolism the most significant pathway changed statistically, while Roberts et al. [331,489] found L-kynurenine and KYNA among the metabolites most raised in terms of predicting both the severity of the disease and poor outcome (implying activation upstream of L-kynurenine as well as of KAT enzymes or transporters). Cihan et al. [490] and Kucukkarapinar et al. [491] reported similar data, while Sindelar and colleagues found KYNA to be the most predictive of disease severity [492]. Cai et al. suggested additional gender differences [493] (though we could not confirm that [331,489]), while L-kynurenine was noted by Almulla and colleagues but not KYNA [494]. Holmes and colleagues [495] inferred elements of the L-kynurenine pathway but reported only ratios. L-kynurenine was also noted by other authors, such as [496,497,498,499,500,501,502] (in these latter cases, KYNA was seemingly not measured, pointing up the utility of untargeted discovery methods for unravelling the biology more fully, both for metabolomics [503] and more generally [504]).

What is a priori unknown, however, for this or other diseases, is the extent to which the upstream metabolites such as L-kynurenine and quinolinic acid, considered to be less beneficial, counteract any possible benefits of KYNA, whether its production represents attempts by the body to use it as a protective agent, or whether it is simply a ‘by-product’ of L-kynurenine due to the presence of KAT activity; this needs testing with KYNA or KATs as an independent variable.

## 14. Protection against Various Diseases

Antioxidants such as ergothioneine are seen as excellent cytoprotectants against multiple stresses [505,506,507,508]. In a similar vein, KYNA has also been demonstrated to be a neuroprotectant and cytoprotectant against a variety of acute challenges. As before with ergothioneine [18], we divide these studies into central and peripheral studies, before looking at reported receptors for KYNA.

### 14.1. Neuroprotection

Many studies, some reviewed recently by [308,461], have indicated the ability of KYNA to serve as a neuroprotective agent, and some of the relevant papers are set out in Table 5.

### 14.2. Peripheral Protection

In Table 6, we summarize some of the examples in which KYNA has proved to be neuroprotective against stresses in non-CNS tissues. Ischemia-reperfusion injury occurs when tissues subjected to hypoxia are reoxygenated, leading to the rapid formation of ROSs; although this is well known in acute circumstances, it is becoming increasingly recognized that this can also occur chronically (e.g., [413]), especially in diseases such as long COVID where fibrinaloid microclots [539,540,541] can induce hypoxia [539] and related sequelae such as reperfusion injury [413] and postural orthostatic tachycardia syndrome (POTS) [542]. As an antioxidant, and probably via other signalling pathways, a particular feature of KYNA is its ability to lower the extent of ischemia-reperfusion injury [517].

Another accompaniment of such diseases is fibrosis and/or amyloid deposition [543,544]. In some cases, fibrin can adopt an amyloid form, e.g., [540,545,546,547,548], though only rarely is fluorescence staining for amyloid performed [549]. Similarly, the ability of KYNA to lower fibrosis [308,550,551,552] may be relevant in this context. 

**Table 6:** Some peripheral disorders in which KYNA has been reported to be protective in mammalian systems.

Organ/Tissue/Disease	Comments	Selected References
Alimentary canal	Protects vs. stress ulcers in rats	[553,554]
Cardiovascular disorders	Review	[555]
Diabetes, type 2	Protective of glomerular filtration rate and against end-stage kidney disease in type 2 diabetes	[556]
Fibrosis	Protective vs. fibrotic injury after surgery	[550,552]
Heart	Protection against ischemia-reperfusion injury	[557,558]
Kidney	Protective of glomerular filtration rate and against end-stage kidney disease in type 2 diabetes	[556]
	Improved kidney function in spontaneously hypertensive and normotensive rats	[559]
Liver	Levels raised in and protective against hexafluoropropylene oxide dimer acid (HFPO-DA) challenge in mice	[560]
	Protection vs. nonalcoholic fatty liver disease at very high concentrations	[561]
Lung	Protective in an acute lung injury model	[562]
Multi-organ	Protection against heatstroke by multiple mechanisms, including an anti-apoptotic effect	[563]
Pancreatitis (acute)	Rat study. Significantly protective at 300 mg/kg	[564]
Retinal ganglia	Protective against ischemia-reperfusion injury in mice	[565]
Sepsis	Protection vs. neutrophil activation and mitochondrial dysfunction in rats	[566]
	Active at high doses against LPS-induced inflammation/death in mice	[567]
Stroke	Associated with a lower level of risk (but probably also confounded with kynurenine); also protective	[568,569,570]
Vascular inflammation	Protective	[571]
Wound healing and scarring	Protective, by largely unknown mechanisms.	[550,552,572,573,574]

### 14.3. Reported Receptors

A number of receptors for KYNA have been detected via ligand binding, albeit often using concentrations far in excess of those measured in vivo. These have recently been reviewed by Turska and colleagues [308], on which Table 7 is partly based. While the data are clear that KYNA can be active at the N-methyl D-aspartate (NMDA) receptor (antagonist), the aryl hydrocarbon receptor (AhR) (agonist), and the GPR35 receptor (agonist), the biological relevance of this awaits an improved understanding of local concentrations of KYNA and other ligands. Meanwhile, the importance of Table 7 for present purposes is more in showing the broad absence of untoward effects at these receptors even when applied concentrations are high.

## 15. Role of KYNA in Protecting against Ischemia-Reperfusion Injury

**Figure 8:**
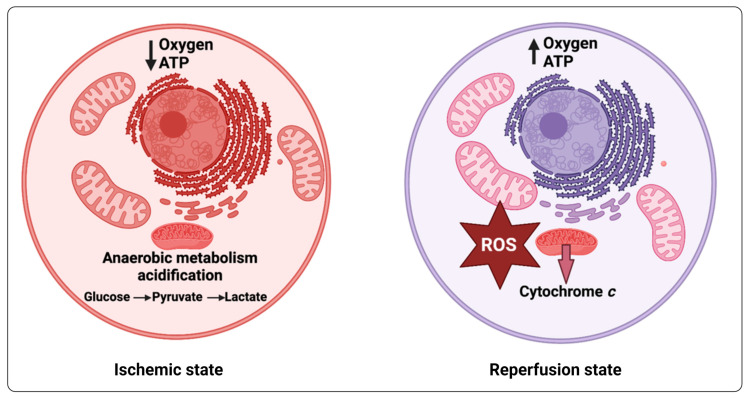
Production of reactive oxygen species as part of ischemia-reperfusion injury. Redrawn from the CC-BY 4.0 paper [413].

Quite a number of the papers mentioned in the above tables highlight a protective role for KYNA in ischemia-reperfusion (I-R) injury, possibly during the hypoxia phase [604]. This I-R injury is a well-known accompaniment [605] in acute circumstances such as stroke [606,607], myocardial infarctions [608,609,610], and organ transplantation [611,612,613,614], as well as in experimental models (e.g., [517,615]). It has recently been recognized as occurring in more chronic circumstances [413] such as long COVID, and as such it is worth highlighting. It occurs when, during a period of hypoxia, commonly caused by ischemia, mitochondria become over-reduced, such that when O_2_ is readmitted (‘perfusion’), it is reduced not with four electrons as normal (to water) by cytochrome oxidase but to peroxide and superoxide by complexes III and I, respectively (Figure 8). These ‘reactive oxygen species’ can react catalytically with unliganded iron molecules (in the Fenton and Haber–Weiss reactions) to produce the especially damaging hydroxyl radical OH^•^ [414], leading to cell death, and the further release of unliganded iron [324], accounting for a number of the symptoms that accompany chronic, inflammatory diseases [410,413]. We consider that analysis of the effects of KYNA on ROS levels and their dynamics is an important and understudied area.

## 16. Other Factors Known to Affect KYNA Levels

As well as adding KYNA directly, other treatments have been found to increase its level. Many of these are seen as beneficial to the host, though the extent to which KYNA contributes is essentially unknown. We exclude those in which known KYNA precursors are simply added explicitly. Table 8 indicates some.

## 17. Other Effects of KYNA

Conversely, a number of papers have studied the effects of KYNA addition on different biochemical pathways; although not exhaustive, the point is to show that they are manifold, and they are summarized in Table 9.

## 18. Safety

Turska and colleagues provide an excellent review [308] of the possibility of enriching foodstuffs with KYNA. By and large, however, human safety studies involving dosing with substantial amounts of pure exogenous KYNA per se have largely not been performed [307], though KYNA (as chestnut honey) was given to human volunteers with no ill effects [306] while a 6 g tryptophan challenge increased KYNA levels more than 130-fold, again without seeming ill-effects, albeit some effects on cerebral blood flow in healthy controls but not in those with schizophrenia-related disorders [664] (rather implying the irrelevance of KYNA here).

Some studies in rodents have added KYNA at massive doses (well over 100 mg/kg, getting into the millimolar range in serum/plasma), seemingly without ill effects (indeed sometimes with protective effects). Table 10 lists some.

We note that Hiratsuka and colleagues [672] gave young female participants up to 5000 mg tryptophan per day (~100 mg/kg based on the stated BMI levels) with no adverse effects; the amount of KYNA that was formed is unknown, though approximately 100 μmol/d (~19 mg) could be found in the urine [672], the value plateauing after ~7 d [673]. Similarly, Al-Karagholi and colleagues [674] dosed volunteers with 5 mg L-kynurenine/kg (say 350 mg total) without apparent ill-effect, while Jauch et al. [675] administered L-kynurenine to rhesus monkeys at doses up to 200 mg/kg, with serum KYNA levels reaching ~25 μM within 10 min, declining to 2.8 μM after 4 h, again without apparent ill effects. Rentschler et al. dosed rats with L-kynurenine at 100 mg/kg, as well as a kynurenine aminotransferase inhibitor at 30 mg/kg; given that on average every marketed drug interacts with at least six known targets [676] (see later section on docking), it is hard to interpret mechanisms during such experiments in which pharmaceutical drugs are added at these kinds of concentration. Note specifically that a hit rate of 1% or more is common in small molecule screens using drug concentrations of just 1–10 μm in individual phenotypic assays (e.g., [258]). L-4-chlorokynurenine is under study as a transportable substrate [677] that can be converted into 7-kynurenic acid, an NMDA receptor antagonist; as part of such studies, doses of L-4-chlorokynurenine of over 1 g per day were well tolerated [678,679]. The main point here is that while any ‘targets’ may remain unknown, there do not seem to be safety issues with these quite substantial doses. 

## 19. Possible Risks

Thus, while the safety profile of KYNA does not yet seem to have been looked at in real detail, we recognize that everyone is different [680], and that there is a tendency for promising studies to become less so over time [681,682,683], not least since possibly ‘occasional’ adverse events are more likely to occur as the populations assessed become larger. We do also note some studies in which KYNA induced possibly undesirable effects. While far less common than those in which it has been shown to be a cytoprotectant, it is appropriate to list some of them (Table 11, see also Table 4). The reasons for such effects are also not really well understood, i.e., whether these are causative or they are essentially downstream responses. As mentioned, the biggest problem with most such studies is that they add tryptophan or L-kynurenine, which can themselves (or their other metabolites) lead to many other undocumented and important changes in host biochemistry. Schizophrenia seems the most common, and as noted, the evidence is at best equivocal as to whether changes in the level of KYNA are a cause or an effect or simply an accompaniment.

## 20. Regulations for Food Supplements and Nutraceuticals

The use of nutraceuticals varies largely from country to country and, like their use, the legislation around such substances vary widely, lack harmonization and are continuously evolving. Comprehensively, some countries as Canada follow stringent regulations, whereas, in some others, as the United States, well-structured and adequate regulations for nutraceuticals are largely absent [685,686]. Regulations for nutraceuticals in different countries of the world are reviewed in [687,688,689].

In the Unites States, nutraceuticals are included in a category called “dietary supplements”, which is regulated under the Dietary Supplement Health and Education Act of 1994 (DSHEA) through the Food and Drug Administration (FDA). The DSHEA states that a dietary supplement must be intended for ingestion and cannot be designated for other use. However, unlike the stricter FDA’s regulations for drugs, the regulations for dietary supplements are more flexible. The manufacturers and distributors are responsible for nutraceutical safety evaluations and correct labelling, while the FDA limits its role to take action against a product that does not follow the requirements of the DSHEA purely after it reaches the market.

All the dietary supplements that were not marketed before the DSHEA are considered and termed “new dietary ingredient”, and while there is no official list of those that were marketed before the Act, the manufacturer is responsible for determining if a substance is a “new dietary ingredient”. In the case of a “new dietary ingredient”, the manufacturer has to notify the FDA about the ingredient at least 75 days before the product goes into the market; providing information (i.e., any citation to published articles) regarding the expected safety of the new ingredient.

On the other hand, the European Union (EU) has more strict regulations for nutraceuticals completed by the European Food Safety Authority (EFSA), which made a comprehensive assessment of substances that are allowed as a source of such molecules in the EU, including safety of the nutrient source, intake levels and bioavailability. According to the EU General Food Law Regulation (EC) No 178/2002, nutraceuticals are considered foodstuffs, therefore regulated as such. The Directive 2002/46/EC established consensus lists of the vitamins and minerals that can be used for manufacturing food supplements and the labelling requirements for these products, while the use of substances other than vitamins or minerals is subject to the laws prevailing in the different Member States.

In case of foodstuffs not consumed in the EU before 1997, the EFSA requires an application for authorization of a “novel food”, including detailed information about composition, intended use, and safety data before the ingredient is released on the market and it can only be commercialized once authorized. Additionally, according to the Regulation (EC) No 2015/2283, EFSA should provide a scientific opinion on a substance’s safety when it undergoes an application for “novel foods”.

## 21. Analytics

Since the coining of the term in 1998 [690], metabolomics studies are well into their third decade [691], where the analysis of small molecules such as KYNA is now dominated by methods combining gas or liquid chromatography with mass spectrometry (e.g., [692,693]). As an aromatic amino acid that ionizes reasonably well, a variety of such analytical methods have indeed been developed (e.g., [307,339,380,381,694,695,696,697,698,699]). In addition, as a redox-active fluorophore (whose fluorescence is enhanced by zinc ions [40,700]), KYNA may also be detected by electrochemistry [35,356] and optically [352,700,701,702,703,704,705,706,707,708,709,710], or (as any organic molecule) by vibrational spectroscopy [711].

## 22. KYNA as a Therapeutic for Chronic Inflammatory Diseases

Many of the diseases mentioned above share similar properties, including in particular that they are chronic and accompanied by inflammation (an interesting recent suggestion around the latter based on mitochondrial antipathy to their cellular host is worth flagging [712]). Thus, any nutraceuticals that might be able to tackle inflammation would be of value, and we have set out here the evidence that leads us to suppose that KYNA might be one. This said, there are seemingly some chronic inflammatory diseases, such as rheumatoid disease [713], that seem not to have major changes in KYNA.

## 23. Use of KYNA as an Antioxidant in Processed Foodstuffs

Although ergothioneine has been used successfully in this way as an antioxidant, e.g., in seafoods [714,715,716], we are not aware of any attempts to use KYNA in this way. We note, however, the important work on the suggestions of fortifying artificial baby milk with it [359] on the grounds that its levels are significantly lower than those of natural human milk.

## 24. KYNA in the Feed of Racing Animals

It is implicit that if KYNA is a nutraceutical, it may have value for elite athletes as well as for the ‘normal’ population. Purified KYNA is unlikely to be economically competitive as an additive in the feed of food animals, but its addition may well be worthwhile for those involved in horse or camel racing. However, the analysis of tryptophan metabolism in such animals is in its infancy [717].

## 25. Use of KYNA in Cosmetics

‘Cosmeceuticals’ are nutraceuticals that are marketed for their cosmetic benefits (e.g., [718,719,720,721,722,723,724]). Because significant skin damage is caused by UV-mediated ROS production [725,726,727,728], it is reasonable—much as with ergothioneine [18]—that KYNA might be useful as a cosmeceutical, and it can both be formulated in creams and taken up into the body [551]. As with ergothioneine, it is possible that its relative unavailability is holding back such uses here, though we note that it is also a photosensitizer. Its value as a topical treatment in inhibiting scarring has, however, been demonstrated [550,573], and Aryl hydrocarbon receptor agonists (of which KYNA is one, and including the FDA-approved tapinarof [729,730]) have been shown to have benefits in both psoriasis and atopic dermatitis [731,732,733,734,735]. Thus, KYNA would seem well worth exploring as a cosmeceutical ingredient.

## 26. Role of KYNA as a Cofactor

While KYNA is clearly capable of acting as an antioxidant directly (as can ergothioneine), most small molecules (including vitamins) interact with proteins of various kinds, and many more than we usually credit (e.g., [736,737,738,739,740]. In addition, the relatively low concentrations of KYNA in humans also imply a more regulatory role that can lead to genetic induction or repression and thus amplification of their signal. In this vein, as our ‘index’ antioxidant nutraceutical, ergothioneine acts in part via the redox-active transcription factor Nrf2 (e.g., [741,742,743,744,745,746]). Studies in KYNA are far behind, but a tantalizing report [747] shows that chestnut honey—the foodstuff containing by far the largest amount of KYNA (Table 3)—can exert protective effects via Nrf2 on LPS-treated macrophages and indomethacin-treated gastric mucosa. Indeed, high levels of KYNA can induce Nrf2 synthesis [94] and prevent the induction of reactive oxygen species [95] and other changes [748] caused by quinolinic acid. While the aryl hydrocarbon receptor AhR is also a transcription factor, its activation can itself stimulate the activation of Nrf2 [732,733,749], and a variety of known agonists target both ArH and Nrf2 [731,750] and assist with atopic dermatitis and psoriasis (see above), so this is reasonable. On the other hand it is activated by dioxins, with somewhat negative effects [749,751], and has a complex expression profile in certain tumors [752]. Its expression is also affected by NF-κB [752,753], a transcription factor whose activity depends on frequency rather than amplitude [754,755,756], and this is still rarely recorded. Deconvolving the detailed interactions between KYNA and AhR is thus a highly non-trivial process.

## 27. Cheminformatics of KYNA

**Figure 9:**
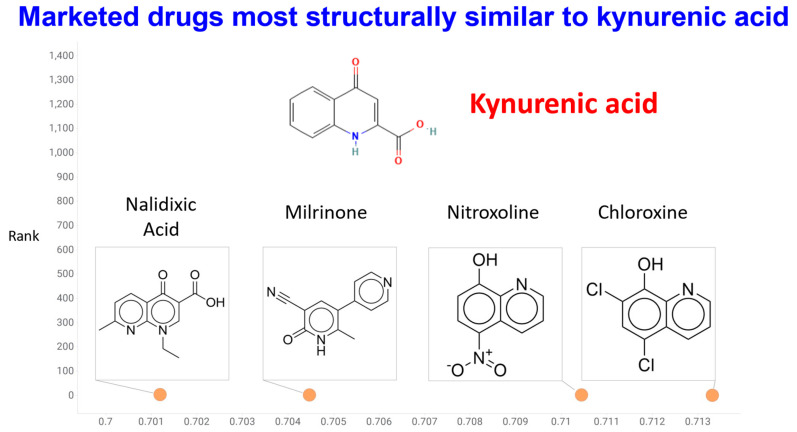
Cheminformatic analysis of the structural similarity of kynurenic acid to those marketed drugs for which the Tanimoto similarity with the RDKit Pattern encoding exceeds 0.7.

One strategy for understanding the biology of a small molecule is to assess how close it is to other endogenous metabolites and, in particular, to marketed drugs, as knowledge of their binding partners or mode of action might give clues to the binding partners of KYNA. One paper shows such an analysis using the RDKit [757]. Pattern encoding and the otherwise precise methods are described in detail elsewhere [310,758,759,760,761]. Only four marketed drugs have a Tanimoto similarity exceeding 0.7, and these are displayed in Figure 9. Interestingly, nalidixic acid is transported in *E. coli* via the fadL fatty acid transporter [762] (not studied in [763]), so this kind of observation may provide clues. However, since this is not our present focus, we simply set out these data and thoughts to guide future studies.

## 28. Predicting the KYNA Interactome

**Figure 10:**
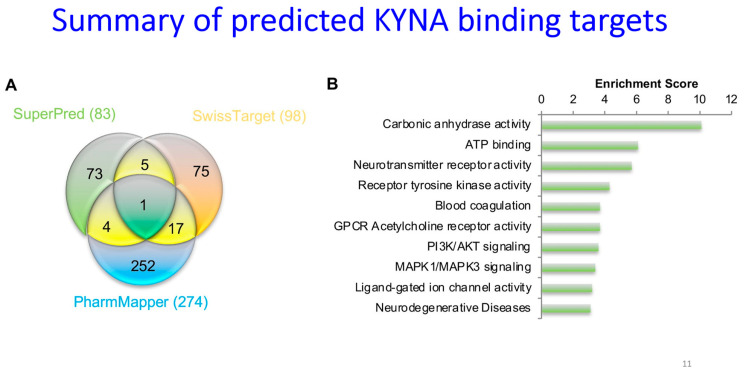
In silico prediction of the KYNA interactome. (**A**). Venn diagram analysis of predicted KYNA interacting proteins from the SuperPred [769], SwissTargetPrediction [768] and PharmMapper [767] cheminformatic tools. (**B**). Functional enrichment analysis of all (n = 455) predicted KYNA interactors. The DAVID [773] knowledgebase was used to identify the top functional annotation clusters, significantly enriched among the predicted KYNA interactome.

Multiple cheminformatic tools now exist for compound–target interaction prediction in silico (e.g., [764,765]) (we ignore those based on generative AI, as they are still in their infancy [766], though this is changing rapidly, and most seek molecules that bind to specified targets, not the other way round as we are interested in here). These tools employ a variety of network-based approaches, machine-learning models, and molecular-docking algorithms to predict the binding of small molecules to target proteins or receptors. As prediction results depend on both the underlying Knowledgebases and the computational approaches applied, it is prudent to examine both the intersection and the compiled results, including with pathway topology and other functional annotation approaches from multiple prediction tools. We have investigated potential KYNA binding targets using three such OpenSource tools with recently updated databases and differing prediction approaches. Specifically, we used PharmMapper [767] that uses a reverse pharmacophore mapping approach and requires a 3D structure (mol2 or sdf format) for ligand input, SwissTargetPrediction [768] that examines 2D and 3D molecular similarity, and SuperPred 3.0 [769] that uses machine learning models for prediction. SwissTargetPrediction and SuperPred take ligand input in a simplified molecular-input line-entry system (SMILES) [770] format. The full dataset is given in Supplementary Spreadsheet S1. Remarkably, only one protein was predicted by all three tools, the thyroid hormone receptor alpha (THA, Figure 10A). This is especially interesting, as previously impaired removal of KYNA from the brain during has been observed in experimental hypothyroidism [625] (Table 8). Twenty-seven predicted targets for KYNA were identified by at least two of these computational compound-target prediction tools, including the aforementioned AhR (Table 12). Other nuclear receptor family members predicted in addition to THA and AhR, included the peroxisome proliferator-activated receptor alpha (PPAR-alpha) and estrogen receptor beta (ER-beta). The latter is notable in the light of a recent report of lower plasma KYNA levels in users of estrogen contraceptives [632] (Table 8). Notably, however, several of those in Table 7 were not picked up using this approach. Also of interest is that KYNA was predicted to bind multiple isomers of the zinc-containing enzyme carbonic anhydrase, which seems at least plausible as tryptophan has been shown by crystallography to bind and activate carbonic anhydrase 2 [771]. Not least of note is the prediction that KYNA was predicted to bind the lymphocyte specific tyrosine kinase (LCK). Critical to T cell signalling, LCK function has been shown by us to be exquisitely sensitive to dietary zinc supply [772], and this too may contribute to the immunological effects caused by KYNA.

As experimentally validated and/or plausible targets were among the proteins predicted by only one tool (e.g., acetylcholine receptors from SuperPred, SLC16A1 from SwissTargetPrediction, albumin from PharmMapper) we performed functional enrichment analyses in the DAVID Knowledgebase [773] on all (*n* = 455) predicted interactors (Figure 10B). The top functional clusters included carbonic anhydrase activity and multiple neuromodulatory receptor and ion channel activities. Notably, neurotransmitter function and neurodegenerative disease were among the top biological functional clusters associated with the predicted KYNA interactome (Figure 10B).

## 29. Biotechnological Production

Current laboratory [774,775,776] and commercial production of KYNA is via chemical synthesis, which presently uses some environmentally unpleasant chemicals and has modest yields. However, as with ergothioneine (e.g., [18,777,778,779]), it is possible to product KYNA by fermentation, Studies of the fermentative production of KYNA are relatively limited, however, and are summarized in Table 13.

## 30. Conclusions and Forward Look

Thanks to advances in scientific knowledge and in public health, human lifespan has been increasing in developed countries since the middle of the 19th century at something like 6 y per 25 y (ungendered and aggregated data for the UK at https://www.statista.com/statistics/1040159/life-expectancy-united-kingdom-all-time/, accessed on 19 August 2024), and its variation between individuals and countries has also decreased [786]. However, the healthspan, the period in which one is free of significant ill-health, has not matched it [787]. It is widely recognized (e.g., [788,789,790]) that diet has a significant role in bringing the healthspan closer to the lifespan. In particular, nutraceuticals, molecules that influence health positively and that might be part of or added to a ‘healthy diet’, are seen as a contributor. Ergothioneine (ERG), a potent and effective antioxidant, seems to be one [1,18,29,508,791,792], with experimental evidence and ongoing studies for this continuing to emerge (e.g., [320,793]), and we here make the case that KYNA is another. It is instructive to provide a comparison of the two molecules, and Table 14 does so.

Overall, the evidence that KYNA may indeed be a worthwhile nutraceutical that might be added exogenously involves at least the following:

- Many examples in which its exogenous addition seems to offer benefits of health or of protection against disease
- Evidence that its concentration is relatively low in normal populations
- Safety evidence to the effect that there do not seem to be examples in which hyperactive alleles of KAT enzymes lead to overt disease, and that exogenous KYNA cannot realistically ‘go back’ to L-kynurenine
- Evidence that it is more or less readily bioavailable for entering plasma from the diet rather than simply being produced by compounds such as tryptophan and L-kynurenine that are more easily transported but that can lead to other, potentially toxic molecules.

This said, not least by comparison with ergothioneine, there are considerable gaps in our knowledge of its biology. In the case of ergothioneine, there has been a massive upsurge in interest in the last 20 years, and as pointed out by Halliwell and Cheah [792], “a key factor was the discovery that an organic cation transporter, OCTN1, is responsible for uptake of “ergothioneine” from the gastrointestinal tract and for its distribution to tissues in the bodies of humans and other animals” (see [802,803]). Given the evidence for ‘missing’ transporters that we present above, a similar trajectory seems plausible for KYNA. 

From the perspective of nutrition, the number of foodstuffs for which KYNA contents have been measured by multiple laboratories using modern, quantitative methods is rather limited, and such studies demand extension.

Other glaring gaps in our knowledge involve pharmacokinetic studies of KYNA uptake, distribution, metabolism and excretion in both humans and laboratory animals (for ergothioneine, see e.g., [319]), the effects of KYNA supplementation on measures of health such as antioxidant status and indeed longevity, and other studies manipulating KYNA as an independent variable to establish suitable nutraceutical dosing levels. We may also be sure that it interacts with other proteins whose identity has not yet been discovered, not least for some of the other targets that were predicted by in silico docking (Table 12), where modern proteomics approaches to attack such questions are available [740,811,812].

The biggest issue with many studies where, for example, tryptophan or L-kynurenine was added, is that they often infer effects of KYNA that are equally plausibly due to changes in other metabolites of the kynurenine pathway or elsewhere that were not in fact measured. In a sense, this is one of the great strengths of KYNA as a candidate nutraceutical, as it can be added without being expected to affect upstream metabolites significantly, at least directly. This offers particular levels of safety.

## Figures and Tables

**Table 1:** Some known substrates/inhibitors of ABCC4 (MRP4) that may assist in raising intracellular and tissue levels of KYNA.

Molecule	Comments	Selected References
Ceefourin-1	Highly selective inhibitor of ABCC4	[271,272,273]
Leukotrienes B_4_/C_4_	K_m_ 0.1–5.6 μM	[274,275,276]
MK-571 (Verlukast)	Inhibitor. Also a quinoline with a carboxylate group. Commonly more potent than probenecid. Also inhibits MRP1.	[266,277,278,279]
Probenecid	Inhibits multiple transporters. K_i_ for SLC22A6/8~15 μM	[280,281,282]
Quercetin	K_i_ 1 μM (and other related polyphenols)	[283]
Reviews		[251,284,285,286]
Sulindac	K_i_ 2 μM	[287]
Urate	Pertinent in gout and kidney disease	[263]

**Table 2:** Some values for measured concentrations of KYNA in human body fluids, and rodent and human tissues.

Serum		
Concentration Range	Comments	References
42 nM median	Approximately doubled in severe, acute COVID-19	[331]
42 nM (35–54 nM IQR)	Increase with age noted	[332]
47 nM median	Marginally lower in ADHD	[333]
38 nM mean	Unchanged by inflammatory bowel diseases	[334]
10-60 nM		[335]
60 nM median	Lower in ALS (ca 40 nM)	[336]
23 nM	No different in pre-eclampsia	[337]
38 nM	Higher in erythrocytes of Parkinson’s disease, implying synthesis or concentration	[338]
28 nM average	Insignificantly lower in depression	[339]
Mean 25 nM, 16 nM in gestational diabetes (GD)	L-kynureine was higher in the GD individuals	[340]
100 nM	Maternal serum; 3–4-fold higher in cord blood	[341]
**Plasma**		
**Concentration range**	**Comments**	**References**
40 nM	15% decrease in migraine	[342]
~30 nM		[343]
~40 nM	No effect in depression	[344]
30–40 nM	Small increase with ibuprofen	[345]
~40 nM	Increased 63% after endurance exercise (150 km road race)	[215]
44 nM median	No change in migraines	[346]
10–80 nM	Median 38 nM; no difference in bipolar individuals	[347]
39–54 nM median	Slightly greater with age and male gender	[348]
~50 nM	Increased 3-fold when SLC22A6/8 inhibited by addition of probenecid	[349]
18–350 nM	Pregnant women, 18–20 weeks, NB concentrated 20-fold in cord blood	[294]
4–60 nM	A summary of multiple measurements	[350]
23 nM mean		[351]
~20 nM	Chinese population	[352]
70 nM in controls, 104 nM in those with social anxiety disorder	Increased with age in controls, but no relation with age in social anxiety disorder	[353]
Mean 21 nM, 23 nM in women with pre-eclampsia (PE)	Strongly influenced by BMI, that may have been a confounder; raised level suggested as a response to the PE rather than a cause	[354]
**CSF**		
**Concentration range**	**Comments**	**References**
~20 nM in control	Increased 3-fold in Alzheimer’s	[355]
1–4 nM	Medan 38 nM; no difference in bipolar individuals	[336]
5 nM	Stable post mortem	[356]
2–5 nM	Strong positive correlation with age	[357]
1–50 nM	Can be raised strongly by certain alleles of KAT II	[358]

**Table 3:** Some sources of kynurenic acid in certain foodstuffs.

Vegetable	Comments	Selected References
Broccoli	0.41 mg/kg	[307]
Chestnut honey	129–601 mg/kg	[392]
Chestnut honey	~400 mg/kg	[393]
Chestnut honey	Up to 2000 mg/kg	[308]
Flower honeys (various)	0.1–2 mg/kg	[392]
Herbs of various kinds	Dandelion leaves 0.5 mg/kgww St John’s wort 32 μg/dose	[386]
Horseshoe crab extract	1200 mg/kg (0.12%)	[360]
Potato tubers	0.1–3.2 mg/kg	[307]
	0.3–3 mg/kg across 16 cultivars	[391]
	3× greater in purple potatoes	[37]
	Review	[308]

**Table 4:** Some of the diseases or syndromes in which normal levels of KYNA are altered. Based in part on the data in [86], and see also the reviews [102,423].

Disease or Syndrome	Source, and Raised or Lowered	Selected References
Acute liver failure	Raised in rat brain as a consequence, via increase in kynurenine in the periphery	[424]
Alzheimer’s dementia	Plasma one third lower Plasma marginal effect	[343,355]
	Serum lower	[425]
	CSF lower	[328,426]
	CSF higher, but seemingly protective vs. disease progression	[427]
	Review	[428]
Aortic stiffness	Correlation of KYNA levels in patients with atrial fibrillation	[429]
Attention deficit hyperactivity disorder (ADHD)	Significantly lowered (meta-analysis of 650 individuals)	[430]
Bipolar depression	~40% decreased vs. controls	[431]
	Review; very variable, mostly lower	[432,433]
Cancers	Very heterogeneous. Inhibition of proliferation observed at very high doses.	[434,435,436]
Cluster headaches and migraines	Serum ~one third lower	[437,438]
COVID-19	Raised in serum, especially in more severe acute cases	Reviewed by [439,440], and see next section
Familial Mediterranean fever	KYNA decreased	[441]
Frailty	Lowered in frailty or little change	[329,442]
Huntington’s disease	Cerebral cortex—four-fold reduction. One molecule (laquinimod) targeting the aryl hydrocarbon receptor in clinical trials	[443,444,445]
Inflammatory bowel disease	Seen as a protective mechanism via raised levels of KATs	[334,446]
Irritable bowel syndrome	Lowered in serum	[447]
	Lowered in urine	[448]
Kidney disease	Normal serum level of 28 nM increased to 336 nM in renal insufficiency	[449]
	Significantly raised in non-survivors of septic shock with acute kidney injury	[450]
	Chronic kidney disease, plasma levels can exceed 500 nM, along with high levels of other tryptophan metabolites	[263,451]
	End-stage kidney disease, plasma levels can exceed 1 μM	[263]
	Significantly raised in renal failure	[452]
Major depressive disorder	30% reduced over healthy controls; seen as the only metabolic biomarker that is both diagnostic and predictive. ~40% decreased over controls Antidepressant activity in mice (at high concentrations) Considered to be related to poor Western diet	[431,453,454,455]
	Lowered in cortex, raised in serum of rats undergoing chronic restraint stress	[456]
Migraine	15% lower KYNA/KYN halved	[342,457]
	Significantly lower, acting via multiple receptors	[458]
Multiple sclerosis	Plasma 45 → 77 nM	[459]
	Erythrocytes 38 → 63 nM	[459]
	Raised in relapsing-remitting MS, lowered in primary and secondary progressive. Quinolinic acid seems to be the real culprit here.	[460,461]
	Lowered in CSF vs. other neurological diseases	[462]
Myalgic encephalopathy/chronic fatigue syndrome (ME/CFS)	Significantly lowered in some tissues, raised in others	[463,464,465]
Osteoporosis	Potential for treatment	[466]
Parkinson’s disease (Review [467])	Frontal cortex—~one third of controls	[468]
	Plasma	[338]
	Serum	
	Substantia nigra—less than half that of controls	[468]
	CSF lower	[328]
Polycystic kidney disease	Significantly raised	[469]
Polycystic ovary syndrome	Roughly doubled	[470]
Pre-eclampsia	Very nonlinear, but significantly raised (especially in those with high BMI) in one Norwegian birth cohort study	[354]
	No effect in a variety of other studies reviewed in:	[337,471]
Pulmonary arterial hypertension	Raised, as were a great many other L-kynurenine pathway metabolites	[472]
Schizophrenia and bipolar disorder	Raised significantly (though usually so are other molecules such as L-kynurenine (which may be the real effector and/or changed by inhibitors of KATII) Lowered in some studies Meta-analysis implies no real or obvious difference	[196,198,199,433,473,474,475,476,477,478,479]
Sjögren’s syndrome	KYNA somewhat raised	[480]
Systemic lupus erythematosus (Lupus)	Many kynurenine pathway metabolites raised	[481]
Type 1 diabetes	Steptozotocin-induced diabetes in rats led to a modest increase in KYNA	[482]
Type 2 diabetes	Raised from 36 to 46 nM	[483]
	Said to be raised during progression	[484]
Ulcerative colitis	Raised, seen as likely coming from changes in microbial gut metabolism. Protection considered to be via GPR35	[377,485]

**Table 5:** Ability of KYNA to serve as a neuroprotective agent.

System	Comments	Selected References
Anticonvulsants	Possible model of mediation via KYNA	[509,510,511]
Depression	Considered neuroprotective in depression	[512]
Epileptic spasms	Lower KYNA in epileptic spams that in other non-inflammatory neurological diseases	[513]
Excitotoxic challenges	KYNA protective	[514,515]
Experimental autoimmune encephalomyelitis	Protective against a Th17 response	[379]
Ischemia-reperfusion	Gerbil brain. Massive doses led to very high intracerebral concentrations of KYNA and neuroprotection	[516]
	Highly protective in a model of hypoxic ischemia in neonatal rats	[517,518]
	Kynurenine sulphate produces KYNA that is neuroprotective in gerbils and rats	[519,520]
Memory enhancement	Effective at lower doses in mice Opposite effect in *C. elegans*	[521,522]
Migraine	Seems to be protective via inhibition of glutaminergic neurons	[523,524,525,526]
Multiple sclerosis	Considered protective	[527,528]
	Plasma 45 → 77 nM	[459]
	Erythrocytes 38 → 63 nM	[459]
Pain	Protective against neuropathic pain, and enhances effectiveness of morphine	[529]
	Antinociceptive in inflammatory pain	[530]
Reviews		[531,532,533]
Spinal cord injury (SCI)	Protective (with glucosamine) against SCI in rats	[534]
Stroke	Mostly protective if given ahead of experimental stroke. Naturally protective against death after adjusting for inflammation Higher levels associated with better recovery	[432,535,536]
Traumatic brain injury	KYNA is overexpressed, attenuates this in rats	[537,538]

**Table 7:** Receptors to which KYNA has been suggested or found to bind.

Putative Receptor	Comments	Selected References
Adrenoceptor alpha 2B (ADRA2B)—Putative ligand	Note that guanfacine is an FDA-approved agonist, used successfully vs. attention deficit hyperactivity disorder (and interestingly also vs. hypertension [575])	[576,577]
	Review	[578]
	Identified in a high-throughput CRISPR screen	[579]
Aryl hydrocarbon receptor (AhR)—Agonist	Induces various pathways, including IL-6 production, at 100 nM	[580,581]
	Protects against intestinal *C. albicans* infection via AhR	[582]
	Possible role in COVID-19	[439,583]
	Protective against acute lung injury	[562]
	Removing AhR raises KYNA levels, and these are neuroprotective against excitotoxic insults	[93]
	KYNA affect neural plasticity via AhR in zebrafish	[584]
	Involved in fibrosis and skin disease	[585] [586]
G-protein-coupled receptor 35 (GPR35)—Agonist	Regulates energy metabolism and ablates weight gain on high-fat diet in mice	[92,213,360,587]
	Protection against ischemic injury (at 5 mg/kg, ≡26 μM if homogeneous)	[588]
	Reviews	[589,590,591]
Glutamate receptor (GAR)—antagonist	Many papers relating to migraines, e.g.,	[523,524,525,526]
Hydroxycarboxylic acid receptor 3 (HCAR3)—putative ligand	Identified in a high-throughput CRISPR screen	[579]
N-methyl D-aspartate (NMDA) receptor (especially the strychnine-sensitive glycine-binding site) (involved in pain [592])—antagonist	EC_50_ 7 μM though binding curves and very complex effects	[69,593,594]
	Potential role in nutritional signalling	[595]
	10 nM can affect differentiation of cortical cells	[596]
	Electrophysiological effects observable at high concentrations	[597]
	Reviews	[598,599,600]
alpha-7 nicotinic acetylcholine receptor (α7nAChR)—purported antagonist	Electrophysiological effects not observed even at high concentrations	[597]
	Active at 7 μM in hippocampal neurons	[601]
	No physiological effects observed with KYNA	[602]
	Lowers inflammatory cytokine production and Abeta phagocytosis	[603]

**Table 8:** Some circumstances in which exogenous elements affect the levels of KYNA.

Substance	Comments	Selected References
Amphetamines	Significant decrease after dosing with amphetamine	[616]
Anthocyanins (in blackberry extract)	Microbiome said to be responsible for increasing KYNA levels (though in the LC-MS data neither the apparent retention time nor the reported mass of the positive molecular ion (*m*/*z* = 208; true is 190) underlying this is that of KYNA)	[617]
Antidiabetic agents	Glibenclamide and metformin both decrease KYNA levels, likely by different mechanisms	[618]
Exercise	Exercise can stimulate the production of katG enzymes and thereby raise KYNA (and lower central L-kynureine), making KYNA an ‘exerkine’. Data are somewhat mixed	[619,620,621]
	KAT4 was especially strongly stimulated in endurance exercise, leading in some cases to more than a 60% increase in plasma KYNA levels	[215]
	Exercise increases KYNA and its activation of the AhR receptor	[622]
Fasting	8 day fasting increased KYNA levels. Effect observable even at 2 days	[623,624]
Hypothyroidism	Experimentally induced hypothyroidism leads to brain KYNA levels being raised	[625]
Insulin signalling	Tested in *C. elegans*, caused increases in KYNA	[522]
Interferon-γ	Massive increase in KYNA in neurons and astrocytes	[626,627]
Ketone bodies	β-hydroxybutyrate stimulates KYNA synthesis and provides neuroprotection	[514,515,628,629]
LPS treatment	Lowers brain KYNA	[630]
Obesity	Higher serum levels of several KP metabolites, including KYNA; in some cases, may simply reflect higher fluxes from raised dietary intake	[631]
Estrogens (as oral contraceptive agents)	Decreased from a median of 58 → 33 nM in plasma	[632]
	Progesterone partial reverses interferon-γ-induced decrease in KYNA	[633]
*Hericium erinaceus* polysaccharides	Hepatoprotective vs. non-alcoholic fatty liver disease, and increase KYNA levels	[634]
Stress	Stress increases KYNA levels in rats, who show lower cognitive skills; unclear whether KYNA response is causally involved or an attempt to counteract. KYNA was not added independently Hepatic KYNA lowers anxiety-induced stress	[635,636]

**Table 9:** Some other known biochemical and physiological effects of KYNA.

Pathway	Comments	Selected References
Amyloid (Aβ) fibrillation and toxicity in *C. elegans*	Inhibited by KYNA, as it was by some simple analogues	[637]
Anti-inflammatory	Includes effects on histone methylation	[638]
	Reverses effects of LPS in macrophage cultures	[639]
	Lowers inflammatory phagocytic response in mouse macrophages	[640,641]
	Lowers LPS-induced inflammation	[642]
	Lowers experimentally induced inflammation in the trigeminal ganglion	[643]
	Hydrogels containing KYNA lowers experimentally induced inflammation	[644]
Anxiolytic (reduces anxiety)	Notable effects in zebrafish at 105 μM (20 mg/L)	[645]
Apoptosis	Induced by KYNA	[646]
	270 genes differentially expressed after exposure to 0.25 mM KYNA	[647]
Astrocyte activation	Inhibitory and protects against HIV-induced cognitive loss	[648]
DNA excision repair	KYNA increases pathway transcription	[649]
Endothelial damage (induced by homocysteine)	Protective	[650]
	Enhances endothelial adhesion and spreading	[651]
Fibroblast growth factor release from HUVEC cells	Inhibited at “low” concentrations (1 μM) of KYNA, while proliferation rate increased.	[652]
Glutamate release	Lowered by KYNA	[653]
Hypertension	Heart rate lowered by KYNA in spontaneously hypertensive rats	[654]
Indoleamine 2,3-dioxygenase induction	Signalling role	[562,655]
Insulin resistance	Protective in high-fat-diet-induction model	[656]
Interleukins	Lowered IL17/IL23 at high concentrations	[657]
Mitochondrial induction	Acts as a cardioprotective	[557]
Neprilysin	Neprilysin degrades amyloids, and KYNA induces its synthesis and is neuroprotective	[658]
TNF-α production	Decreased at very high concentrations	[659,660,661]
Unfolded protein response	KYNA inhibits, and is protective in a *C. elegans* Alzheimer’s model	[662]
Vasculature	Induces vascular relaxation in endothelial cells	[663]

**Table 10:** Some studies in which organisms or mammalian cells have been exposed to exceptionally high concentrations of KYNA.

Organism	Dose and Comment	Selected References
Gerbils	400–1600 mg/kg; protected against ischemia-reperfusion injury	[516]
Mice	250 mg/L in drinking water, ≡25 mg/kg/d, has no toxic effects	[640,641]
	25–250 mg/L drinking water 3–21 d; well tolerated.	[665]
Rat	500 mg/kg i.p.—protected against thioacetamide-induced liver injury	[666]
	300 mg/kg i.p. in young rats prolonged wakefulness	[323,667]
	150 mg/kg lowers morphine-conditioned reward behavior	[668]
	25 mg/kg/d in drinking water ≡ ~250 mg/L, assists healthy growth in rat babies	[359]
	300 mg/kg i.p. protect against mussel toxin	[554]
	200 mg/kg kynuramine	[669]
	300 mg/kg vs. acute pancreatitis	[564]
	300 mg/kg in young rats was neuroprotective	[517,520]
	As much as 5% of diets, with large KYNA excretion, small decrease in weight gain, but seemingly without major ill effects.	[670,671]
**Mammalian cell lines**		
Murine RAW 264.7 macrophages	100 μM KYNA is protective against LPS challenges	[639]
Murine BV-2 microglial cells	No effect on viability of 100 μM KYNA	[603]
Rat splenocytes	No effects on viability of proliferation at 500 μM	[641]
Humans	Topical application; no safety issues observed. Most secreted in urine	[551]

**Table 11:** Some examples in which KYNA was considered to have some potentially negative effects.

General Biological Area	Comments	Selected References
Cardiovascular disease	Marginally associated with (a 10% increase) in all-cause, but likely confounded with L-kynurenine that is more significant	[568]
Schizophrenia	KYNA levels often raised, though unclear if this is a cause or an attempted detoxification of raised L-kynurenine. Some studies showed them lowered; overall unclear. See also the text.	[196,473,474,476,478,684]

**Table 12:** KYNA protein targets predicted by two or more computational cheminformatics tools.

Protein Names	UniProt ID	Entry Name	Predicted to Bind KYNA by
Thyroid hormone receptor alpha	P10827	THA_HUMAN	SuperPred, SwissTarget, PharmMapper
Glutathione S-transferase P	P09211	GSTP1_HUMAN	SuperPred, PharmMapper
Inosine-5′-monophosphate dehydrogenase 2	P12268	IMDH2_HUMAN	SuperPred, PharmMapper
Galectin-3	P17931	LEG3_HUMAN	SuperPred, PharmMapper
Histone deacetylase 8	Q9BY41	HDAC8_HUMAN	SuperPred, PharmMapper
Gamma-aminobutyric acid receptor subunit alpha-1	P14867	GBRA1_HUMAN	SuperPred, SwissTarget
D-amino-acid oxidase	P14920	OXDA_HUMAN	SuperPred, SwissTarget
Amine oxidase (flavin-containing) A	P21397	AOFA_HUMAN	SuperPred, SwissTarget
Aryl hydrocarbon receptor	P35869	AHR_HUMAN	SuperPred, SwissTarget
Dual specificity protein kinase CLK4	Q9HAZ1	CLK4_HUMAN	SuperPred, SwissTarget
Prothrombin Thrombin heavy chain	P00734	THRB_HUMAN	SwissTarget, PharmMapper
Renin	P00797	RENI_HUMAN	SwissTarget, PharmMapper
Carbonic anhydrase 1	P00915	CAH1_HUMAN	SwissTarget, PharmMapper
Carbonic anhydrase 2	P00918	CAH2_HUMAN	SwissTarget, PharmMapper
Thymidylate synthase	P04818	TYSY_HUMAN	SwissTarget, PharmMapper
Lymphocyte specific tyrosine kinase	P06239	LCK_HUMAN	SwissTarget, PharmMapper
Neprilysin	P08473	NEP_HUMAN	SwissTarget, PharmMapper
Leukotriene A-4 hydrolase	P09960	LKHA4_HUMAN	SwissTarget, PharmMapper
Thyroid hormone receptor beta	P10828	THB_HUMAN	SwissTarget, PharmMapper
Angiotensin-converting enzyme	P12821	ACE_HUMAN	SwissTarget, PharmMapper
Farnesyl pyrophosphate synthase	P14324	FPPS_HUMAN	SwissTarget, PharmMapper
Neutrophil collagenase	P22894	MMP8_HUMAN	SwissTarget, PharmMapper
Macrophage metalloelastase	P39900	MMP12_HUMAN	SwissTarget, PharmMapper
Aldo-keto reductase family 1 member C3	P42330	AK1C3_HUMAN	SwissTarget, PharmMapper
Mitogen-activated protein kinase 10	P53779	MK10_HUMAN	SwissTarget, PharmMapper
Peroxisome proliferator-activated receptor alpha	Q07869	PPARA_HUMAN	SwissTarget, PharmMapper
Estrogen receptor beta (ER-beta)	Q92731	ESR2_HUMAN	SwissTarget, PharmMapper

**Table 13:** Fermentative production of KYNA in microorganisms.

Organism	Genetic Modification(s)	Titer	Conditions and Comments	References
*Escherichia coli*	Remove competing pathway, enhance SAM pathway	350 mg/mL	Main target was actinocin	[780]
*Saccharomyces cerevisiae*	None, but a chemical defined medium including 400 mg/L tryptophan was used	9 mg/L		[781]
		~1.5 μM (~280 ng/mL)		[782]
*Yarrowia lipolytica*	None	21 μg/mL in culture broth or 494 μg/g cell dry weight	trp-supplemented media	[783,784,785]

**Table 14:** A comparison of some properties ergothioneine and KYNA in terms of our knowledge and their present status as nutraceuticals.

Property	Ergothioneine	Kynurenic Acid
Overall status as a nutraceutical	Fairly well established [1,18,29,508,791,792,794,795]	Emerging [86,99,308,533]
Biosynthesized endogenously	No	Yes
Largest dietary source	Mushrooms (fruiting bodies of basidiomycetes) [29,796,797,798,799]	Chestnut honey [308,392]
Highest natural product level	~9 g/kg [29,800]	~0.6 g/kg [308,392]
Approximate serum/plasma level in ‘healthy’ populations	~260 ng/mL, ~1.1 μM	~40 nM
Degree of concentration in erythrocytes	Maybe as much as 10 times [801]	Possibly 3× but unclear how calculated [425]; no increase in a related study [459]
Known ‘uptake’ transporters in humans	Relatively specific and concentrative e.g., SLC22A4 [802,803], SLC22A15 [804]	Those known are non-specific and non-concentrative [235,236]
Concentrated in erythrocytes as a kind of ‘buffer’ for plasma	Significantly (up to ten-fold [801,805]), probably accumulated via SLC22A4 [806]	Much less so (note that RBC express ABCC4 [807])
Known ‘efflux’ (pump) transporters in humans	Not known	ABCC4 [242,696] and possibly ABCG2 [263,264]
Known transporters in microorganisms	Several, e.g., [778,808,809]	None seemingly published
Thermostability	High (can be extracted at 95 °C [810])	High (stable to boiling and frying [37]), and in blood samples [699]
Pharmacokinetics studies	Somewhat, with human feeding trials for 35 d [319]	Not really initiated orally

## Supplementary Materials

The following supporting information can be downloaded at: https://www.mdpi.com/article/10.3390/ijms25169082/s1.
