# Supplementary material for: The Biology and Biochemistry of Kynurenic Acid, a Potential Nutraceutical with Multiple Biological Effects
Source: Int J Mol Sci. 2024 Aug 21;25(16):9082. doi: 10.3390/ijms25169082 (PMC11354673; doi:10.3390/ijms25169082)
Supplement: Supplementary file 1 [file ijms-25-09082-s001.zip › ijms-3139788-supplementary/Supplementary Table S1.pdf]

Supplementary Table S1 Part 1. Kinetic parameters of the Tryptophan and Indoleamine Dioxygenases

Substrate	k_{cat} (s^{-1})	K_M (µM)	k_{cat}/K_M (M^{-1} s^{-1})	K_D (mM)	References
Tryptophan 2,3-dioxygenase					
L-Trp	1.4 ± 0.019	222 ± 15	6.31E+03	0.17 ± 0.011(Fe^{3+})[c]	(Basran et al., 2008; Batabyal & Yeh, 2007; Lewis-Ballester et al., 2016)
D-Trp	0.073[a]	ND	NC		
5-fluoro-Trp	0.18 ± 0.004	360 ± 28	5.00E+02		
5-methyl-Trp	ND	ND	0.0001(µM^{-1} s^{-1})[b]		
5-hydroxy-Trp	NA				
N-methyl-Trp	NA				
tryptamine	NA				
indole-3-propionic acid	NA				
tryptophanol	NA				
Indoleamine 2,3-dioxygenase 1					
L-Trp	2.97 ± 0.20	20.90 ± 3.95	1.42E+05	0.32 ± 0.03 (Fe^{3+})[c]	(Basran et al., 2008; Pantouris et al., 2014; Sugimoto et al., 2006)
D-Trp	2.7 ± 0.3	296 ± 19	9.12E+03	0.53 ± 0.05 (Fe^{2+})[c]	
1-Me-L-Trp	0.062 ± 0.001	70 ± 1	8.86E+02		
1-Me-D-Trp	0.095 ± 0.007	660 ± 43	1.44E+02		
5-F-D,L-Trp	0.76 ± 0.01	6 ± 1	1.27E+05		
5-Me-D,L-Trp	3.78 ± 0.16	98 ± 14	3.86E+04		
5-HO-L-Trp	0.025 ± 0.0004	17 ± 1	1.47E+03		
N-methyl-Trp	NA				
tryptamine	NA				
indole-3-propionic acid	NA				
tryptophanol	NA				
Indoleamine 2,3-dioxygenase 2					
L-Trp	0.103 ± 0.006	6,809 ± 917	15.13		(Pantouris et al., 2014)
D-Trp	0.0103 ± 0.0006	3,609 ± 675	2.85		
1-Me-L-Trp	0.011 ± 0.006	696 ± 41	15.80		
1-Me-D-Trp	0.0052 ± 0.0004	747 ± 168	6.96		
5-F-D,L-Trp	0.049 ± 0.002	1,768 ± 161	27.71		
5-MeO-D,L-Trp	0.161 ± 0.003	547 ± 29	294.33		
5-Me-D,L-Trp	0.36 ± 0.02	1,570 ± 188	229.30		
6-Me-D,L-Trp	0.108 ± 0.013	3,457 ± 962	31.24		
melatonin	NA				
5-HO-L-Trp	NA				

[a]at 50 mM of D-Trp

[b]initial velocity was linearly dependent on 5-methyl-Trp concentration across the experimental range

[c]for L-Trp

ND - not demonstrated

NC - not calculated

NA - no activity

Supplementary Table S1 Part 2. Kinetic parameters of the Kynurenine formamidase

Substrate	V_{max} (mM/min)	K_M (mM)	References
Kynurenine formamidase/aryl formamidase (Afmid)[a]			
N-Formyl-L-kynurenine	60	0.19 ± 0.01	(Pabarcus & Casida, 2005)

[a]kinetic parameters obtained from mouse (*Mus musculus*) KF (68.5% similarity and identity with the human KF – calculated using the Sequence Manipulation Suite (https://bioinformatics.org/sms2/ident_sim) and the Uniprot Q8K4H1 and Q63HM1 as inputs)

Supplementary Table S1 Part 3. Kinetic parameters of the Kynurenine Aminotransferases

Substrate	Co-substrate	k_{cat} (min^{-1})	K_M (mM)	k_{cat}/K_M (mM^{-1} min^{-1})	References
Kynurenine aminotransferase I/Glutamine transaminase/Cysteine conjugate β-lyase (KAT-1)					
Glutamine	α-ketobutyrate	440.5 ± 28.7	2.8 ± 0.5	157.3	(Han et al., 2004, 2009b; Okuno et al., 1991)
Phenylalanine		91.0 ± 4.8	1.7 ± 0.3	53.5	
Leucine		339.9 ± 43.1	7.6 ± 3	44.7	
Kynurenine		201.1 ± 19.2	4.7 ± 0.4	42.8	
Tryptophan		43.1 ± 3.4	1.2 ± 0.3	35.9	
Methionine		215.4 ± 14.4	6.4 ± 0.9	33.7	
Tyrosine		91.0 ± 4.8	3.2 ± 0.4	28.4	
Histidine		143.6 ± 14.4	5.4 ± 1	26.6	
Cysteine		9.6 ± 0.48	0.7 ± 0.1	13.7	
Amino-butyrate		38.3 ± 4.8	21.3 ± 4.7	1.8	
Asparagine		14.4 ± 0.14	23.1 ± 5.7	0.6	
L-kynurenine	α-Ketoleucine	296.8 ± 28.7	1 ± 0.3	247.4	
	Glyoxylate	263.3 ± 28.7	1.5 ± 0.5	175.5	
	Phenylpyruvate	110.1 ± 19.2	0.8 ± 0.4	137.6	
	Mercaptopyruvate	234.6 ± 14.4	2.5 ± 0.4	93.8	
	α-Ketobutyrate	234.6 ± 9.6	3 ± 0.4	78.2	
	Oxaloacetate	143.6 ± 9.6	4.2 ± 0.4	34.2	
	Pyruvate	28.7 ± 4.8	12.1 ± 4.9	2.4	
Kynurenine aminotransferase II/α-aminoadipate aminotransferase (KAT-2)					
Aminoadipate	glyoxylate	179.4 ± 5.7	0.9 ± 0.1	196.2	(Guillemin et al., 2007; Han et al., 2008; Okuno et al., 1991)
Kynurenine		585.3 ± 39.9	4.7 ± 0.8	125.9	
Methionine		206.4 ± 17	1.7 ± 0.5	123.8	
Glutamate		185.0 ± 14.8	1.6 ± 0.4	118.7	
Tyrosine		131.5 ± 5.2	1.8 ± 0.2	74.4	
Phenylalanine		327.1 ± 46.2	5.2 ± 1.7	63.4	
Tryptophan		254.0 ± 8.6	4.3 ± 0.4	58.7	
Leucine		285.0 ± 70.4	5.1 ± 3.0	56.1	
3-hydroxy-kynurenine		102.1 ± 12.2	3.8 ± 1.0	26.8	
Glutamine		95.5 ± 16.6	8.1 ± 2.9	11.8	
Alanine		173.6 ± 43.2	19.4 ± 9.2	9	
Aminobutyrate		157.2 ± 19.4	17.8 ± 4.3	8.8	
L-Kynurenine	α-Oxoglutarate	460.1 ± 49.8	1.2 ± 0.4	374.5	
	α-Oxocaproic acid	289.7 ± 8.1	1.5 ± 0.1	188	
	Phenylpyruvate	284.0 ± 13	1.8 ± 0.2	156.9	
	α-oxo-γ-methiol-butyric acid	256.2 ± 31.9	2.4 ± 0.7	105.2	
	Mercaptopyruvate	215.5 ± 12.6	2.8 ± 0.5	77.7	
	Indo-3-pyruvate	89.1 ± 32.5	1.4 ± 1.2	64.6	
	α-Oxovalerate	159.3 ± 4.9	3.4 ± 0.3	47	
	α-Oxoleucine	150.5 ± 13.6	3.3 ± 0.8	45.4	
	α-Oxobutyrate	208.9 ± 12.5	12.7 ± 2.1	16.4	
	Hydroxy-phenylpyruvate	23.6 ± 9.5	1.5 ± 1.1	16.2	
	α-Oxoadipate	290.3 ± 30	20.9 ± 5	13.9	
	Glyoxylate	218.4 ± 18.4	18 ± 3.7	12.1	

	Oxaloacetate	93.9 ± 51.6	16.8 ± 12.4	5.6	
	α-Oxovaline	55.1 ± 4.6	12.9 ± 2.9	4.3	
	α-Oxoisoleucine	55.2 ± 6.9	14.2 ± 3.8	3.9	
	Pyruvate	21.8 ± 6.4	9.7 ± 5.6	2.3	
Kynurenine aminotransferase III/cysteine conjugate beta-lyase 2 (KAT-3)[a]					
Glutamine		136.0 ± 14.0	0.7 ± 0.2	194.2	(Han et al., 2009a, 2010; Yu et al., 2006)
Histidine		120.0 ± 20.0	0.7 ± 0.4	171.4	
Methionine		146.0 ± 50.0	0.9 ± 0.7	162.2	
Phenylalanine		162.0 ± 20.0	1.1 ± 0.4	147.2	
Asparagine		176.0 ± 18.0	1.4 ± 0.4	125.7	
Cysteine	glyoxylate	78.0 ± 12.0	0.7 ± 0.4	111.4	
Kynurenine		138.0 ± 18.0	1.5 ± 0.5	92	
Serine		130.0 ± 10.0	3.0 ± 0.7	43.3	
Tryptophan		213.9 ± 60.0	7.1 ± 4.2	30.1	
Tyrosine		62.0 ± 8.0	2.7 ± 0.9	23	
Alanine		92.0 ± 21.9	6.2 ± 2.5	14.8	
	Glyoxylate	162.8 ± 35.4	0.4 ± 0.2	407	
	α-Ketocaproic acid	155.3 ± 16.2	0.5 ± 0.2	310.6	
	Phenylpyruvate	152.0 ± 27.3	0.6 ± 0.3	253.3	
	α-Ketobutyrate	175.5 ± 7.4	1.0 ± 0.1	175.5	
	α-oxo-γ-methiol-butyric acid	60.9 ± 11.9	0.4 ± 0.2	152.3	
	α-Ketovalerate	162.2 ± 11.5	1.2 ± 0.3	135.27	
L-kynurenine	Indo-3-pyruvate	62.0 ± 13.0	0.5 ± 0.5	124	
	Hydroxy-phenylpyruvate	88.9 ± 5.0	1.0 ± 0.1	88.9	
	Mercaptopyruvate	196.5 ± 13.6	2.4 ± 0.5	81.9	
	Oxaloacetate	220.7 ± 27.6	4.9 ± 1.2	45	
	Pyruvate	112.3 ± 16.9	10.6 ± 3.8	10.6	
	α-Ketoisocaproic acid	44.4 ± 6.3	5.3 ± 1.4	8.4	
	α-Ketoglutarate	22.2 ± 11.7	8.1 ± 12.8	2.7	
Kynurenine aminotransferase IV/aspartate aminotransferase/ glutamate-oxaloacetate aminotransferase (KAT-4)[b]					
	Phenylpyruvate	37.7 ± 6.9	0.7 ± 0.4	57.8	(Han et al., 2010, 2011)
	Oxaloacetate	19.2 ± 2.6	0.9 ± 0.4	21.1	
	Hydroxyphenylpyruvate	24.7 ± 5.5	1.6 ± 0.7	15.7	
	α-Ketoglutarate	32 ± 2.7	2.4 ± 0.6	13.4	
	Mercaptopyruvate	26.1 ± 1.6	3.2 ± 0.7	8.1	
	Indo-3-pyruvate	29.1 ± 1.1	3.6 ± 0.3	8	
L-kynurenine	α-oxo-γ-methiol-butyric acid	24.8 ± 0.7	5.7 ± 0.5	4.3	
	Glyoxylate	11.4 ± 1.1	4.2 ±0.8	2.7	
	Pyruvate	22.1 ± 0.9	8.3 ± 1.0	2.7	
	α-Ketocaproic acid	24.6 ± 1.2	10.4 ± 1.4	2.4	
	α-Ketobutyrate	41.3 ± 7.9	42.2 ± 14.2	1	
	α-Ketovalerate	7.5 ± 0.4	10.9 ± 1.5	0.7	

[a]kinetic parameters obtained from mouse (*Mus musculus*) KAT-3 (86.8% similarity and 83.7% identity with the hKAT-3)

[b]kinetic parameters obtained from mouse (*Mus musculus*) KAT-4 (94.6% identity and 97.4% similarity with the hKATIV – calculated using the Sequence Manipulation Suite (https://bioinformatics.org/sms2/ident_sim) and the Uniprot P05202 and P00505 as inputs)

References to Supplementary Table 1

Basran, J., Rafice, S. A., Chauhan, N., Efimov, I., Cheesman, M. R., Ghamsari, L., & Raven, E. L. (2008). A kinetic, spectroscopic, and redox study of human tryptophan 2,3-dioxygenase. *Biochemistry*, *47*(16), 4752–4760. https://doi.org/10.1021/bi702393b

Batabyal, D., & Yeh, S. R. (2007). Human tryptophan dioxygenase: A comparison to indoleamine 2,3-dioxygenase. *Journal of the American Chemical Society*, *129*(50), 15690–15701. https://doi.org/10.1021/ja076186k

Guillemin, G. J., Cullen, K. M., Lim, C. K., Smythe, G. A., Garner, B., Kapoor, V., Takikawa, O., & Brew, B. J. (2007). Characterization of the kynurenine pathway in human neurons. *Journal of Neuroscience*, *27*(47), 12884–12892. https://doi.org/10.1523/JNEUROSCI.4101-07.2007

Han, Q., Cai, T., Tagle, D. A., & Li, J. (2010). Thermal stability, pH dependence and inhibition of four murine kynurenine aminotransferases. In *BMC Biochemistry* (Vol. 11). http://www.biomedcentral.com/1471-2091/11/19

Han, Q., Cai, T., Tagle, D. A., Robinson, H., & Li, J. (2008). Substrate specificity and structure of human aminoadipate aminotransferase/kynurenine aminotransferase ii. *Bioscience Reports*, *28*(4), 205–215. https://doi.org/10.1042/BSR20080085

Han, Q., Li, J., & Li, J. (2004). pH dependence, substrate specificity and inhibition of human kynurenine aminotransferase I. *European Journal of Biochemistry*, *271*(23–24), 4804–4814. https://doi.org/10.1111/j.1432-1033.2004.04446.x

Han, Q., Robinson, H., Cai, T., Tagle, D. A., & Li, J. (2009a). Biochemical and Structural Properties of Mouse Kynurenine Aminotransferase III. *Molecular and Cellular Biology*, *29*(3), 784–793. https://doi.org/10.1128/mcb.01272-08

Han, Q., Robinson, H., Cai, T., Tagle, D. A., & Li, J. (2009b). Structural insight into the inhibition of human kynurenine aminotransferase I/Glutamine transaminase K. *Journal of Medicinal Chemistry*, *52*(9), 2786–2793. https://doi.org/10.1021/jm9000874

Han, Q., Robinson, H., Cai, T., Tagle, D. A., & Li, J. (2011). Biochemical and structural characterization of mouse mitochondrial aspartate aminotransferase, a newly identified kynurenine aminotransferase-IV. *Bioscience Reports*, *31*(5), 323–332. https://doi.org/10.1042/BSR20100117

Lewis-Ballester, A., Forouhar, F., Kim, S. M., Lew, S., Wang, Y., Karkashon, S., Seetharaman, J., Batabyal, D., Chiang, B. Y., Hussain, M., Correia, M. A., Yeh, S. R., & Tong, L. (2016). Molecular basis for catalysis and substrate-mediated cellular stabilization of human tryptophan 2,3-dioxygenase. *Scientific Reports*, *6*. https://doi.org/10.1038/srep35169

Okuno, E., Nakamura, M., & Schwarcz, R. (1991). Two kynurenine aminotransferases in human brain. In *Brain Research* (Vol. 542).

Pabarcus, M. K., & Casida, J. E. (2005). Cloning, expression, and catalytic triad of recombinant arylformamidase. *Protein Expression and Purification*, *44*(1), 39–44. https://doi.org/10.1016/j.pep.2005.04.013

Pantouris, G., Serys, M., Yuasa, H. J., Ball, H. J., & Mowat, C. G. (2014). Human indoleamine 2,3-dioxygenase-2 has substrate specificity and inhibition characteristics distinct from those of indoleamine 2,3-dioxygenase-1. *Amino Acids*, *46*(9), 2155–2163. https://doi.org/10.1007/s00726-014-1766-3

Sugimoto, H., Oda, S. I., Otsuki, T., Hino, T., Yoshida, T., & Shiro, Y. (2006). Crystal structure of human indoleamine 2,3-dioxygenase: Catalytic mechanism of O2 incorporation by a heme-containing dioxygenase. *Proceedings of the National Academy of Sciences of the United States of America*, *103*(8), 2611–2616. https://doi.org/10.1073/pnas.0508996103

Yu, P., Li, Z., Zhang, L., Tagle, D. A., & Cai, T. (2006). Characterization of kynurenine aminotransferase III, a novel member of a phylogenetically conserved KAT family. *Gene*, *365*(1-2 SPEC. ISS.), 111–118. https://doi.org/10.1016/j.gene.2005.09.034
