# Supplementary material for: The Biology and Biochemistry of Kynurenic Acid, a Potential Nutraceutical with Multiple Biological Effects
Source: Int J Mol Sci. 2024 Aug 21;25(16):9082. doi: 10.3390/ijms25169082 (PMC11354673; doi:10.3390/ijms25169082)
Supplement: Supplementary file 1 [file ijms-25-09082-s001.zip › ijms-3139788-supplementary/Supplementary Table S2.pdf]

Supplementary Table S2. Part 1. Non-exhaustive list of human and mouse tissue distribution of enzymes involved in KYNA synthesis

Organism	Cell type/ Tissue distribution[c,d]	Expression	References
Tryptophan 2,3-dioxygenase			
human[a]	liver[d]	constitutive	(Opitz et al., 2011)
	dermal fibroblasts[c]	constitutive	(Asp et al., 2011a)
	brain (prefrontal cortex and cerebellum)[c]	constitutive	(Afia et al., 2021)
	brain (neurons)[c,d]	constitutive	(Guillemin et al., 2007; Miller et al., 2004)
mouse[b]	testis, spermatozoa[d]	constitutive	(Britan et al., 2006a)
	spleen, lymph nodes[c]	constitutive, low expression	(Lanz et al., 2017)
	brain[c,d]	constitutive	(Lanz et al., 2017)
	liver[d]	constitutive, expression increased during inflammation in a model of multiple sclerosis	(Lanz et al., 2017)
	skeletal muscle[c]	constitutive, inducible by chronic stress	(Agudelo et al., 2014a)
	uterus[d]	early pregnancy stages	(Li et al., 2014; Tatsumi et al., 2000)
	concept (early gestation), placenta[c,d]	early pregnancy stages - days 5.5 to 10.5 (preceeds IDO1 expression)	(Suzuki et al., 2001a)
Indoleamine 2,3-dioxygenase 1			
human	placenta[c,d]	constitutive - increases at term (38 - 40 weeks)	(Karahoda et al., 2020)
	neurons[c,d]		(Guillemin et al., 2007)
	lungs[d]	constitutive	(Theate et al., 2015)
	duodenum, small bowel, colon[d]	constitutive	(Theate et al., 2015)
	uterus, fallopian tube[d]	constitutive	(Theate et al., 2015)
	lymph node[d]	constitutive	(Theate et al., 2015)
	spleen[d]	constitutive	(Theate et al., 2015)
	thymus[d]	constitutive	(Theate et al., 2015)
	tonsils[d]	constitutive	(Theate et al., 2015)
	Peyes's patch[d]	constitutive	(Theate et al., 2015)
	brain (prefrontal cortex and cerebellum)[c]	constitutive	(Afia et al., 2021)
	corneal fibroblasts and epithelial cells[d]	induced by IFN-y	(Ryu & Kim, 2007)
	β-cells and non-endocrine cells (pancreatic islets)[d]	constitutive and inducible by IFN-y	(Sarkar et al., 2007)
	macrophages[c,d]	induced by IFN-y	(Croitoru-Lamoury et al., 2011; Favennec et al., 2015a; Munn et al., 1999)
	myeloid dendridic cells[d]	induced by PGE_2	(Trabanelli et al., 2014)

	eosinophils[d]	constitutive and inducible by IFN-y	(Odemuyiwa et al., 2004)
	mesenchymal stem cells[c,d]	induced by IFN-y	(Croitoru-Lamoury et al., 2011; Meisel et al., 2004)
	adipocytes[c]	constitutive and increased expression by IFN-y	(Favennec et al., 2015a)
	astrocytes[c]	constitutive and increased expression by IFN-y	(Croitoru-Lamoury et al., 2011)
	dermal fibroblasts[c]	induced by IFN-γ	(Asp et al., 2011a)
	peripheral blood mononuclear cells[c]	constitutive and increased after exercising	(Joisten et al., n.d.-a)
	decidual glandular epithelium, endothelium of fetal blood vessels (human-fetal interface)[d]	inducible by IFN-y	(Kudo et al., 2020)
mouse	kidney[d]	constitutive	(Ball et al., 2007)
	placenta[c,d]	not expressed in early embryonic stages, but highly expressed from pregnancy day 7.5 to 12.5	(Suzuki et al., 2001b)
	corneal endothelial cells[d]	inducible by TNF and IFN-y	(Beutelspacher et al., 2006)
	epididymis[d]	constitutive	(Britan et al., 2006b)
	skeletal muscle[c]	constitutive, inducible by chronic stress, L-kynurenine	(Agudelo et al., 2014a)
	dendridic cells[d]	constitutive	(Fallarino et al., 2002)
	macrophages[d]	inducible by IFN-y	(Fallarino et al., 2002)
	mesenchymal stem cells[c,d]	induced by IFN-y	(Croitoru-Lamoury et al., 2011)
Indoleamine 2,3-dioxygenase 2			
human	myeloid and plasmacytoid dendridic cells[d/c]	constitutive/ inducible by IFN-y	(Lob et al., 2008; Trabanelli et al., 2014)
	placenta[c]	constitutive	(Kudo et al., 2020)
	syncytiotrophoblast (human-fetal interface)[d]	constitutive	(Kudo et al., 2020)
	lungs[d]	constitutive	(Mandarano et al., 2020)
	peripheral blood mononuclear cells[c]	constitutive and increased after exercising	(Joisten et al., n.d.-a)
	mesenchymal stem cells[c]	induced by IFN-y and INF-β	(Croitoru-Lamoury et al., 2011)
	macrophages[c]	expression reduced in presence of IFN-y	(Croitoru-Lamoury et al., 2011)
	brain (prefrontal cortex and cerebellum)[c]	constitutive	(Afia et al., 2021)
	astrocytes[c]	constitutive	(Croitoru-Lamoury et al., 2011)
mouse	kidney[c,d]	constitutive	(Ball et al., 2007; Jusof et al., 2017)

	testis[c], epididymis (spermatozoa)[d]	constitutive	(Ball et al., 2007; Fukunaga et al., 2012)
	skeletal muscle[c]	constitutive, inducible by chronic stress	(Agudelo et al., 2014a)
	liver[d]	constitutive	(Jusof et al., 2017)
	cerebral cortex, cerebellum[d]	constitutive	(Fukunaga et al., 2012)

[a]*Homo sapiens*

[b]*Mus musculus*

[c]mRNA level

[d]protein level

Supplementary Table S2. Part 2. Non-exhaustive list of human and mouse tissue distribution of enzymes involved in KYNA synthesis

Organism	Cell type/ Tissue distribution[c,d]	Expression	Reference
Kynurenine formamidase/aryl formamidase (Afmid)			
human[a]	adipocytes[c]	constitutive	(Favennec et al., 2015a)
	macrophages[c]	constitutive	(Favennec et al., 2015a)
mouse[b]	liver[d]	constitutive	(Schuettengruber et al., 2003)
	kidney[d]	constitutive	(Schuettengruber et al., 2003)
	heart[d]	constitutive	(Schuettengruber et al., 2003)
	skeletal muscle[c]	constitutive	(Agudelo et al., 2014a)

[a]*Homo sapiens*

[b]*Mus musculus*

[c]mRNA level

[d]protein level

Supplementary Table S2. Part 3. Non-exhaustive list of human and mouse tissue distribution of enzymes involved in KYNA synthesis

Organism	Cell type/ Tissue distribution[d,e]	Expression	Reference
Kynurenine aminotransferase I/Glutamine transaminase/Cysteine conjugate β-lyase (KAT-1)			
human[a]	astrocytes[d]	constitutive, inducible by IFN-γ	(Guillemin et al., 2001; Okuno et al., 1991)
	skeletal muscle[d,e]	constitutive, induced after exercising	(Agudelo et al., 2014a; Schlittler et al., 2016; Wyckelsma et al., 2020a)
	placenta[d]	constitutive	(Manuelpillai et al., 2005; Milart et al., 2001)

	cornea[e]	constitutive	(Matysik-Woźniak et al., 2017a)
	dermal fibroblasts[d]	supressed by IFN-γ+TNF-α	(Asp et al., 2011a)
	macrophages[d,e]	inducible by IFN-γ	(Asp et al., 2011a)
	neurons[d]	low expression, constitutive	(Guillemin et al., 2007)
	colon[e]	constitutive	(Walczak et al., 2011a)
	adipocytes[d]	constitutive	(Favennec et al., 2015a)
mouse[b]	kidney[d]	constitutive	(Hoffmann et al., 2021)
	liver[d]	constitutive	(Hoffmann et al., 2021)
	brain[d]	constitutive	(Yu et al., 2006a)
	skeletal muscle[d]	constitutive, inducible by chronic stress	(Agudelo et al., 2014a; Schlittler et al., 2016)
Kynurenine aminotransferase II/α-aminoadipate aminotransferase (KAT-2)			
human	astrocytes[d]	constitutive, inducible by IFN-γ	(Guillemin et al., 2001; Okuno et al., 1991)
	liver[d]	constitutive	(Goh et al., 2002)
	cornea[e]	constitutive	(Matysik-Woźniak et al., 2017b)
	skeletal muscle[d,e]	constitutive, induced after exercising	(Agudelo et al., 2014b; Schlittler et al., 2016; Wyckelsma et al., 2020b)
	macrophages[d,e]	inducible by IFN-γ	(Goh et al., 2002; Guillemin et al., 2007)
	neurons[d]	low expression, constitutive	(Guillemin et al., 2007)
	colon[e]	constitutive	(Walczak et al., 2011b)
	adipocytes[d]	constitutive	(Favennec et al., 2015b)
rat[c]	astrocytes[e]	constitutive	(Guidetti et al., 2007)
mouse	neurons (cerebellum)[e]	constitutive	(Balog et al., 2021)
	brain[d]	constitutive	(Ishida et al., 2018)
Kynurenine aminotransferase III/cysteine conjugate beta-lyase 2 (KAT-3)			
human	skeletal muscle[d,e]	constitutive, induced after exercising	(Agudelo et al., 2014b; Schlittler et al., 2016; Wyckelsma et al., 2020b)
	brain[d]	constitutive	(Ishida et al., 2018; Yu et al., 2006b)
	cornea[e]	constitutive	(Matysik-Woźniak et al., 2017b)
	dermal fibroblasts[d]	supressed by IFN-γ+TNF-α	(Asp et al., 2011b)
	adipocytes[d]	constitutive, inducible by IFN-γ	(Favennec et al., 2015b)
	peripheral blood mononuclear cells[d]	constitutive and increased after exercising	(Joisten et al., n.d.-b)
mouse	kidney[d]	constitutive	(Yang et al., 2016)

	liver[d]	constitutive	(Yang et al., 2016)
	testis[d]	constitutive	(Yu et al., 2006b)
	heart[d]	constitutive	(Yu et al., 2006b)
	brain[d]	constitutive	(Yu et al., 2006b)
	skeletal muscle[d]	constitutive, inducible by chronic stress	(Agudelo et al., 2014b; Schlittler et al., 2016)
Kynurenine aminotransferase IV/aspartate aminotransferase/glutamate-oxaloacetate aminotransferase (KAT-4)			
human	skeletal muscle[d,e]	constitutive, induced after exercising	(Agudelo et al., 2014b; Schlittler et al., 2016)
	dermal fibroblasts[d]	supressed by IFN-γ+TNF-α	(Asp et al., 2011b)
	peripheral blood mononuclear cells[d]	constitutive and increased after exercising	(Joisten et al., n.d.-b)
mouse	brain[d]	constitutive	(Ishida et al., 2018)
	skeletal muscle[d]	constitutive, inducible by chronic stress	(Agudelo et al., 2014b; Schlittler et al., 2016)

[a]*Homo sapiens*

[b]*Mus musculus*

[c]*Rattus norvegicus*

[d]RNA level

[e]protein level

References for Supplementary Table 2

Afia, A. Ben, Vila, È., MacDowell, K. S., Ormazabal, A., Leza, J. C., Haro, J. M., Artuch, R., Ramos, B., & Garcia-Bueno, B. (2021). Kynurenine pathway in post-mortem prefrontal cortex and cerebellum in schizophrenia: relationship with monoamines and symptomatology. *Journal of Neuroinflammation*, *18*(1). https://doi.org/10.1186/s12974-021-02260-6

Agudelo, L. Z., Femenía, T., Orhan, F., Porsmyr-Palmertz, M., Goiny, M., Martinez-Redondo, V., Correia, J. C., Izadi, M., Bhat, M., Schuppe-Koistinen, I., Pettersson, A. T., Ferreira, D. M. S., Krook, A., Barres, R., Zierath, J. R., Erhardt, S., Lindskog, M., & Ruas, J. L. (2014a). Skeletal muscle PGC-1α1 modulates kynurenine metabolism and mediates resilience to stress-induced depression. *Cell*, *159*(1), 33–45. https://doi.org/10.1016/j.cell.2014.07.051

Agudelo, L. Z., Femenía, T., Orhan, F., Porsmyr-Palmertz, M., Goiny, M., Martinez-Redondo, V., Correia, J. C., Izadi, M., Bhat, M., Schuppe-Koistinen, I., Pettersson, A. T., Ferreira, D. M. S., Krook, A., Barres, R., Zierath, J. R., Erhardt, S., Lindskog, M., & Ruas, J. L. (2014b). Skeletal muscle PGC-1α1 modulates kynurenine metabolism and mediates resilience to stress-induced depression. *Cell*, *159*(1), 33–45. https://doi.org/10.1016/j.cell.2014.07.051

Asp, L., Johansson, A.-S., Mann, A., Owe-Larsson, B., Urbanska, E. M., Kocki, T., Kegel, M., Engberg, G., Lundkvist, G. B., & Karlsson, H. (2011a). *Effects of pro-inflammatory cytokines on expression of kynurenine pathway enzymes in human dermal fibroblasts*. http://www.journal-inflammation.com/content/8/1/25

Asp, L., Johansson, A.-S., Mann, A., Owe-Larsson, B., Urbanska, E. M., Kocki, T., Kegel, M., Engberg, G., Lundkvist, G. B., & Karlsson, H. (2011b). *Effects of pro-inflammatory cytokines on expression of kynurenine pathway enzymes in human dermal fibroblasts*. http://www.journal-inflammation.com/content/8/1/25

Ball, H. J., Sanchez-Perez, A., Weiser, S., Austin, C. J. D., Astelbauer, F., Miu, J., McQuillan, J. A., Stocker, R., Jermiin, L. S., & Hunt, N. H. (2007). Characterization of an indoleamine 2,3-dioxygenase-like protein found in humans and mice. *Gene*, *396*(1), 203–213. https://doi.org/10.1016/j.gene.2007.04.010

Balog, E., Jenei, G., Gellért, L., Ono, E., Vécsei, L., Toldi, J., & Kis, Z. (2021). Species-specific neuronal localization of kynurenine aminotransferase-2 in the mouse cerebellum. *Neurochemistry International*, *142*. https://doi.org/10.1016/j.neuint.2020.104920

Beutelspacher, S. C., Pillai, R., Watson, M. P., Tan, P. H., Tsang, J., McClure, M. O., George, A. J. T., & Larkin, D. F. P. (2006). Function of indoleamine 2,3-dioxygenase in corneal allograft rejection and prolongation of allograft survival by over-expression. *European Journal of Immunology*, *36*(3), 690–700. https://doi.org/10.1002/eji.200535238

Britan, A., Maffre, V., Tone, S., & Drevet, J. R. (2006a). Quantitative and spatial differences in the expression of tryptophan-metabolizing enzymes in mouse epididymis. *Cell and Tissue Research*, *324*(2), 301–310. https://doi.org/10.1007/s00441-005-0151-7

Britan, A., Maffre, V., Tone, S., & Drevet, J. R. (2006b). Quantitative and spatial differences in the expression of tryptophan-metabolizing enzymes in mouse epididymis. *Cell and Tissue Research*, *324*(2), 301–310. https://doi.org/10.1007/s00441-005-0151-7

Croitoru-Lamoury, J., Lamoury, F. M. J., Caristo, M., Suzuki, K., Walker, D., Takikawa, O., Taylor, R., & Brew, B. J. (2011). Interferon-γ regulates the proliferation and differentiation of mesenchymal stem cells via activation of indoleamine 2,3 dioxygenase (IDO). *PLoS ONE*, *6*(2). https://doi.org/10.1371/journal.pone.0014698

Fallarino, F., Vacca, C., Orabona, C., Belladonna, M. L., Bianchi, R., Marshall, B., Keskin, D. B., Mellor, A. L., Fioretti, M. C., Grohmann, U., & Puccetti, P. (2002). Functional expression of indoleamine 2,3-dioxygenase by murine CD8α dendritic cells. In *International Immunology* (Vol. 14, Issue 1).

Favennec, M., Hennart, B., Caiazzo, R., Leloire, A., Yengo, L., Verbanck, M., Arredouani, A., Marre, M., Pigeyre, M., Bessede, A., Guillemin, G. J., Chinetti, G., Staels, B., Pattou, F., Balkau, B., Allorge, D., Froguel, P., & Poulain-Godefroy, O. (2015a). The kynurenine pathway is activated in human obesity and shifted toward kynurenine monooxygenase activation. *Obesity*, *23*(10), 2066–2074. https://doi.org/10.1002/oby.21199

Favennec, M., Hennart, B., Caiazzo, R., Leloire, A., Yengo, L., Verbanck, M., Arredouani, A., Marre, M., Pigeyre, M., Bessede, A., Guillemin, G. J., Chinetti, G., Staels, B., Pattou, F., Balkau, B., Allorge, D., Froguel, P., & Poulain-Godefroy, O. (2015b). The kynurenine pathway is activated in human obesity and shifted toward kynurenine monooxygenase activation. *Obesity*, *23*(10), 2066–2074. https://doi.org/10.1002/oby.21199

Fukunaga, M., Yamamoto, Y., Kawasoe, M., Arioka, Y., Murakami, Y., Hoshi, M., & Saito, K. (2012). Studies on tissue and cellular distribution of indoleamine 2,3-dioxygenase 2: The absence of IDO1 upregulates IDO2 expression in the epididymis. *Journal of Histochemistry and Cytochemistry*, *60*(11), 854–860. https://doi.org/10.1369/0022155412458926

Goh, D. L. M., Patel, A., Thomas, G. H., Salomons, G. S., Schor, D. S. M., Jakobs, C., & Geraghty, M. T. (2002). *Characterization of the human gene encoding a-aminoadipate aminotransferase (AADAT)*. www.academicpress.com

Guidetti, P., Hoffman, G. E., Melendez-Ferro, M., Albuquerque, E. X., & Schwarcz, R. (2007). Astrocytic localization of kynurenine aminotransferase II in the rat brain visualized by immunocytochemistry. *GLIA*, *55*(1), 78–92. https://doi.org/10.1002/glia.20432

Guillemin, G. J., Cullen, K. M., Lim, C. K., Smythe, G. A., Garner, B., Kapoor, V., Takikawa, O., & Brew, B. J. (2007). Characterization of the kynurenine pathway in human neurons. *Journal of Neuroscience*, *27*(47), 12884–12892. https://doi.org/10.1523/JNEUROSCI.4101-07.2007

Guillemin, G. J., Kerr, S. J., Smythe, G. A., Smith, D. G., Kapoor, V., Armati, P. J., Croitoru, J., & Brew, B. J. (2001). Kynurenine pathway metabolism in human astrocytes: A paradox for neuronal protection. *Journal of Neurochemistry*, *78*(4), 842–853. https://doi.org/10.1046/j.1471-4159.2001.00498.x

Hoffmann, D., Dvorakova, T., Schramme, F., Stroobant, V., Van den Eynde, B. J., Klaessens, S., Stroobant, V., De Plaen, E., Van den Eynde, B. J., Ishimura, Y., Nozaki, M., Hayaishi, O., Fukumura, E., Sugimoto, H., Misumi, Y., Ogura, T., Shiro, Y., Zhang, Y., Kang, S. A., … Kanaya, S. (2021). Kynurenine aminotransferase 3/glutamine transaminase L/cysteine conjugate beta-lyase 2 is a major glutamine transaminase in the mouse kidney. *Journal of Inflammation*, *8*(1), 1–7. https://doi.org/10.1186/1476-9255-8-25

Ishida, Y., Fujita, H., Aratani, S., Chijiiwa, M., Taniguchi, N., Yokota, M., Ogihara, Y., Uoshima, N., Nagashima, F., Uchino, H., & Nakajima, T. (2018). The NRF2-PGC-1ß pathway activates kynurenine aminotransferase 4 via attenuation of an E3 ubiquitin ligase, synoviolin, in a cecal ligation/perforation-induced septic mouse model. *Molecular Medicine Reports*, *18*(2), 2467–2475. https://doi.org/10.3892/mmr.2018.9175

Joisten, N., Walzik, D., Schenk, A., Metcalfe, A. J., Belen, S., Schaaf, K., Gehlert, S., Spiliopoulou, P., Garzinsky, A.-M., Thevis, M., Rappelt, L., Donath, L., Meuth, S. G., Bloch, W., & Zimmer, P. (n.d.-a). *Acute exercise increases systemic kynurenine pathway metabolites and activates the AHR in human PBMCs*. https://doi.org/10.1101/2024.01.17.576018

Joisten, N., Walzik, D., Schenk, A., Metcalfe, A. J., Belen, S., Schaaf, K., Gehlert, S., Spiliopoulou, P., Garzinsky, A.-M., Thevis, M., Rappelt, L., Donath, L., Meuth, S. G., Bloch, W., & Zimmer, P. (n.d.-b). *Acute exercise increases systemic kynurenine pathway metabolites and activates the AHR in human PBMCs*. https://doi.org/10.1101/2024.01.17.576018

Jusof, F. F., Bakmiwewa, S. M., Weiser, S., Too, L. K., Metz, R., Prendergast, G. C., Fraser, S. T., Hunt, N. H., & Ball, H. J. (2017). Investigation of the tissue distribution and physiological roles of indoleamine 2,3-dioxygenase-2. *International Journal of Tryptophan Research*, *10*. https://doi.org/10.1177/1178646917735098

Karahoda, R., Abad, C., Horackova, H., Kastner, P., Zaugg, J., Cerveny, L., Kucera, R., Albrecht, C., & Staud, F. (2020). Dynamics of Tryptophan Metabolic Pathways in Human Placenta and Placental-Derived Cells: Effect of Gestation Age and Trophoblast Differentiation. *Frontiers in Cell and Developmental Biology*, *8*. https://doi.org/10.3389/fcell.2020.574034

Kudo, Y., Koh, I., & Sugimoto, J. (2020). Localization of Indoleamine 2,3-Dioxygenase-1 and Indoleamine 2,3-Dioxygenase-2 at the Human Maternal-Fetal Interface. *International Journal of Tryptophan Research*, *13*. https://doi.org/10.1177/1178646920984163

Lanz, T. V., Williams, S. K., Stojic, A., Iwantscheff, S., Sonner, J. K., Grabitz, C., Becker, S., Böhler, L. I., Mohapatra, S. R., Sahm, F., Küblbeck, G., Nakamura, T., Funakoshi, H., Opitz, C. A., Wick, W., Diem, R., & Platten, M. (2017). Tryptophan-2,3-Dioxygenase (TDO) deficiency is associated with subclinical neuroprotection in a mouse model of multiple sclerosis. *Scientific Reports*, *7*. https://doi.org/10.1038/srep41271

Li, D. D., Gao, Y. J., Tian, X. C., Yang, Z. Q., Cao, H., Zhang, Q. L., Guo, B., & Yue, Z. P. (2014). Differential expression and regulation of Tdo2 during mouse decidualization. *Journal of Endocrinology*, *220*(1), 73–83. https://doi.org/10.1530/JOE-13-0429

Lob, S., Konigsrainer, A., Schafer, R., Rammensee, H.-G., Opelz, G., & Terness, P. (2008). *Levo-but not dextro-1-methyl tryptophan abrogates the IDO activity of human dendritic cells*. https://doi.org/10.1182/blood-2007-10

Mandarano, M., Bellezza, G., Belladonna, M. L., Vannucci, J., Gili, A., Ferri, I., Lupi, C., Ludovini, V., Falabella, G., Metro, G., Mondanelli, G., Chiari, R., Cagini, L., Stracci, F., Roila, F., Puma, F., Volpi, C., & Sidoni, A. (2020). Indoleamine 2,3-Dioxygenase 2 Immunohistochemical Expression in Resected Human Non-small Cell Lung Cancer: A Potential New Prognostic Tool. *Frontiers in Immunology*, *11*. https://doi.org/10.3389/fimmu.2020.00839

Manuelpillai, U., Ligam, P., Smythe, G., Wallace, E. M., Hirst, J., & Walker, D. W. (2005). Identification of kynurenine pathway enzyme mRNAs and metabolites in human placenta: Up-regulation by inflammatory stimuli and with clinical infection. *American Journal of Obstetrics and Gynecology*, *192*(1), 280–288. https://doi.org/10.1016/j.ajog.2004.06.090

Matysik-Woźniak, A., Jünemann, A., Turski, W. A., Wnorowski, A., Jóźwiak, K., Paduch, R., Okuno, E., Moneta-Wielgoś, J., Chorągiewicz, T., Maciejewski, R., & Rejdak, R. (2017a). *The presence of kynurenine aminotransferases in the human cornea: Evidence from bioinformatics analysis of gene expression and immunohistochemical staining*. http://www.molvis.org/molvis/v23/364

Matysik-Woźniak, A., Jünemann, A., Turski, W. A., Wnorowski, A., Jóźwiak, K., Paduch, R., Okuno, E., Moneta-Wielgoś, J., Chorągiewicz, T., Maciejewski, R., & Rejdak, R. (2017b). *The presence of kynurenine aminotransferases in the human cornea: Evidence from bioinformatics analysis of gene expression and immunohistochemical staining*. http://www.molvis.org/molvis/v23/364

Meisel, R., Zibert, A., Laryea, M., Göbel, U., Däubener, W., & Dilloo, D. (2004). Human bone marrow stromal cells inhibit allogeneic T-cell responses by indoleamine 2,3-dioxygenase-mediated tryptophan degradation. *Blood*, *103*(12), 4619–4621. https://doi.org/10.1182/blood-2003-11-3909

Milart, P., Urbanska, E. M., Turski, W. A., Paszkowski, T., & Sikorski, R. (2001). Kynurenine Aminotransferase I Activity in Human Placenta. *Placenta*, *22*(2–3), 259–261. https://doi.org/10.1053/PLAC.2000.0611

Miller, C. L., Llenos, I. C., Dulay, J. R., Barillo, M. M., Yolken, R. H., & Weis, S. (2004). Expression of the kynurenine pathway enzyme tryptophan 2,3-dioxygenase is increased in the frontal cortex of individuals with schizophrenia. *Neurobiology of Disease*, *15*(3), 618–629. https://doi.org/10.1016/j.nbd.2003.12.015

Munn, D. H., Shafizadeh, E., Attwood, J. T., Bondarev, I., Pashine, A., & Mellor, A. L. (1999). Inhibition of T Cell Proliferation by Macrophage Tryptophan Catabolism. In *J. Exp. Med* (Vol. 189, Issue 9). http://www.jem.org

Odemuyiwa, S. O., Ghahary, A., Li, Y., Puttagunta, L., Lee, J. E., Musat-Marcu, S., Ghahary, A., & Moqbel, R. (2004). Cutting Edge: Human Eosinophils Regulate T Cell Subset Selection through Indoleamine 2,3-Dioxygenase. *The Journal of Immunology*, *173*(10), 5909–5913. https://doi.org/10.4049/jimmunol.173.10.5909

Okuno, E., Nakamura, M., & Schwarcz, R. (1991). Two kynurenine aminotransferases in human brain. In *Brain Research* (Vol. 542).

Opitz, C. A., Litzenburger, U. M., Sahm, F., Ott, M., Tritschler, I., Trump, S., Schumacher, T., Jestaedt, L., Schrenk, D., Weller, M., Jugold, M., Guillemin, G. J., Miller, C. L., Lutz, C., Radlwimmer, B., Lehmann, I., Von Deimling, A., Wick, W., & Platten, M. (2011). An endogenous tumour-promoting ligand of the human aryl hydrocarbon receptor. *Nature*, *478*(7368), 197–203. https://doi.org/10.1038/nature10491

Ryu, Y. H., & Kim, J. C. (2007). Expression of indoleamine 2,3-dioxygenase in human corneal cells as a local immunosuppressive factor. *Investigative Ophthalmology and Visual Science*, *48*(9), 4148–4152. https://doi.org/10.1167/iovs.05-1336

Sarkar, S. A., Wong, R., Hackl, S. I., Moua, O., Gill, R. G., Wiseman, A., Davidson, H. W., & Hutton, J. C. (2007). Induction of indoleamine 2,3-dioxygenase by interferon-γ in human islets. *Diabetes*, *56*(1), 72–79. https://doi.org/10.2337/db06-0617

Schlittler, M., Goiny, M., Agudelo, L. Z., Venckunas, T., Brazaitis, M., Skurvydas, A., Kamandulis, S., Ruas, J. L., Erhardt, S., Westerblad, H., & Andersson, D. C. (2016). Endurance exercise increases skeletal muscle kynurenine aminotransferases and plasma kynurenic acid in humans. *Am J Physiol Cell Physiol*, *310*, 836–840. https://doi.org/10.1152/ajpcell.00053.2016.-Physical

Schuettengruber, B., Doetzlhofer, A., Kroboth, K., Wintersberger, E., & Seiser, C. (2003). Alternate activation of two divergently transcribed mouse genes from a bidirectional promoter is linked to changes in histone modification. *Journal of Biological Chemistry*, *278*(3), 1784–1793. https://doi.org/10.1074/jbc.M204843200

Suzuki, S., Tone, S., Takikawa, O., Kubo, T., Kohno, I., & Minatogawa, Y. (2001a). Expression of indoleamine 2,3-dioxygenase and tryptophan 2,3-dioxygenase in early concepti. In *Biochem. J* (Vol. 355).

Suzuki, S., Tone, S., Takikawa, O., Kubo, T., Kohno, I., & Minatogawa, Y. (2001b). Expression of indoleamine 2,3-dioxygenase and tryptophan 2,3-dioxygenase in early concepti. In *Biochem. J* (Vol. 355).

Tatsumi, K., Higuchi, T., Fujiwara, H., Nakayama, T., Egawa, H., Itoh, K., Fujii, S., & Fujita, J. (2000). Induction of tryptophan 2,3-dioxygenase in the mouse endometrium during implantation. *Biochemical and Biophysical Research Communications*, *274*(1), 166–170. https://doi.org/10.1006/bbrc.2000.3115

Theate, I., Van Baren, N., Pilotte, L., Moulin, P., Larrieu, P., Renauld, J. C., Herve, C., Gutierrez-Roelens, I., Marbaix, E., Sempoux, C., & Van Den Eynde, B. J. (2015). Extensive profiling of the expression of the indoleamine 2,3-dioxygenase 1 protein in normal and tumoral human tissues. *Cancer Immunology Research*, *3*(2), 161–172. https://doi.org/10.1158/2326-6066.CIR-14-0137

Trabanelli, S., Očadlíková, D., Ciciarello, M., Salvestrini, V., Lecciso, M., Jandus, C., Metz, R., Evangelisti, C., Laury-Kleintop, L., Romero, P., Prendergast, G. C., Curti, A., & Lemoli, R. M. (2014). The SOCS3-Independent Expression of IDO2 Supports the Homeostatic Generation of T Regulatory Cells by Human Dendritic Cells. *The Journal of Immunology*, *192*(3), 1231–1240. https://doi.org/10.4049/jimmunol.1300720

Walczak, K., Dąbrowski, W., Langner, E., Zgrajka, W., Piłat, J., Kocki, T., Rzeski, W., & Turski, W. A. (2011a). Kynurenic acid synthesis and kynurenine aminotransferases expression in colon derived normal and cancer cells. *Scandinavian Journal of Gastroenterology*, *46*(7–8), 903–912. https://doi.org/10.3109/00365521.2011.579159

Walczak, K., Dąbrowski, W., Langner, E., Zgrajka, W., Piłat, J., Kocki, T., Rzeski, W., & Turski, W. A. (2011b). Kynurenic acid synthesis and kynurenine aminotransferases expression in colon derived normal and cancer cells. *Scandinavian Journal of Gastroenterology*, *46*(7–8), 903–912. https://doi.org/10.3109/00365521.2011.579159

Wyckelsma, V. L., Lindkvist, W., Venckunas, T., Brazaitis, M., Kamandulis, S., Pääsuke, M., Ereline, J., Westerblad, H., & Andersson, D. C. (2020a). Kynurenine aminotransferase isoforms display fiber-type specific expression in young and old human skeletal muscle. *Experimental Gerontology*, *134*. https://doi.org/10.1016/j.exger.2020.110880

Wyckelsma, V. L., Lindkvist, W., Venckunas, T., Brazaitis, M., Kamandulis, S., Pääsuke, M., Ereline, J., Westerblad, H., & Andersson, D. C. (2020b). Kynurenine aminotransferase isoforms display fiber-type specific expression in young and old human skeletal muscle. *Experimental Gerontology*, *134*. https://doi.org/10.1016/j.exger.2020.110880

Yang, C., Zhang, L., Han, Q., Liao, C., Lan, J., Ding, H., Zhou, H., Diao, X., & Li, J. (2016). Kynurenine aminotransferase 3/glutamine transaminase L/cysteine conjugate beta-lyase 2 is a major glutamine transaminase in the mouse kidney. *Biochemistry and Biophysics Reports*, *8*, 234–241. https://doi.org/10.1016/j.bbrep.2016.09.008

Yu, P., Li, Z., Zhang, L., Tagle, D. A., & Cai, T. (2006a). Characterization of kynurenine aminotransferase III, a novel member of a phylogenetically conserved KAT family. *Gene*, *365*(1-2 SPEC. ISS.), 111–118. https://doi.org/10.1016/j.gene.2005.09.034

Yu, P., Li, Z., Zhang, L., Tagle, D. A., & Cai, T. (2006b). Characterization of kynurenine aminotransferase III, a novel member of a phylogenetically conserved KAT family. *Gene*, *365*(1-2 SPEC. ISS.), 111–118. https://doi.org/10.1016/j.gene.2005.09.034
